# Abundance of clinically relevant antimicrobial resistance genes in the golden jackal (*Canis aureus*) gut

**DOI:** 10.1128/msphere.00819-24

**Published:** 2025-02-13

**Authors:** Roi Lapid, Yair Motro, Hillary Craddock, Ikram Salah, Roni King, Katherine Winner, Gila Kahila Bar-Gal, Jacob Moran-Gilad

**Affiliations:** 1 The Robert H. Smith Faculty of Agriculture, Food and Environment, The Hebrew University of Jerusalem, Rehovot, Israel; 2 Department of Health Policy and Management, School of Public Health, Faculty of Health Sciences, Ben-Gurion University of the Negev, Be'er-Sheva, Israel; 3 Science and Conservation Division, Israel Nature and Parks Authority, Jerusalem, Israel; 4 Division of Pulmonary and Critical Care Medicine, Department of Internal Medicine, University of Michigan Health System, Ann Arbor, Michigan, USA; 5 Department of Microbiology and Immunology, University of Michigan Medical School, Ann Arbor, Michigan, USA; Nanjing University of Chinese Medicine, Nanjing, Jiangsu, China

**Keywords:** qPCR, antimicrobial resistance, metagenomics, fecal microbiota, golden jackal (*Canis aureus)*, One Health

## Abstract

The spread of antimicrobial resistance (AMR) is a critical One Health issue. Wildlife could act as reservoirs or vehicles of AMR bacteria (ARBs) and AMR genes (ARGs) but are relatively understudied. We sought to investigate clinically relevant ARGs in golden jackals (*Canis aureus*) thriving near human settlements in Israel. Fecal samples were collected from 111 jackals across four regions over a 10-month period. Various animal and spatio-temporal metadata were collected. Samples were analyzed by quantitative PCR (qPCR) for beta-lactamases (*bla*TEM, *bla*CTX-M15, and *bla*SHV), *qnrS* and *int1*. A subset of samples was subject to shotgun metagenomic sequencing followed by resistome and microbiome analyses. qPCR detected a high prevalence of ARGs, including beta-lactamases (*bla*TEM-1, 96.4%; *bla*CTX-M-15, 51.4%, *bla*SHV, 15.3%), fluoroquinolone resistance (*qnrS*, 87.4%), and class 1 integrons (*Int1*, 94.6%). The *bla*TEM-1 gene was found to be more prevalent in adult jackals compared to younger ones. Metagenomic analysis of a subset of samples revealed a diverse gut microbiome harboring a rich resistome with tetracycline resistance genes being the most prevalent. Metagenome-assembled genome analysis further identified several ARGs associated with clinically relevant bacteria. These findings highlight the potential role of golden jackals as reservoirs for AMR and emphasize the need for ongoing surveillance to better understand AMR transmission dynamics at the wildlife–human interface.

**IMPORTANCE:**

The research highlights the potential role of the golden jackals as reservoirs for antimicrobial resistance (AMR). The high prevalence of clinically relevant AMR genes in these jackals emphasizes the need for ongoing surveillance and monitoring to better understand AMR transmission dynamics at the wildlife–human interface.

## INTRODUCTION

The “One Health” paradigm, which integrates the health of humans, animals, and the environment, is a critical framework to view antimicrobial resistance (AMR), given the possibility of human-animal transmission of AMR (1). In the context of the “One Health” approach, AMR is mainly investigated in humans and in domesticated animals, while wildlife remains relatively understudied (2–4). Wildlife can be exposed to AMR bacteria (ARB) and AMR genes (ARGs) through human waste (including sewage and food wastes) and animal products (i.e., livestock feces and feed) (5, 6) or via contact with domesticated animals. Wildlife may potentially act as a vehicle or reservoir for resistant bacteria across borders (transboundary movement) and continents (4, 7, 8).

Scavengers and synanthropic species can acquire and spread AMR due to their feeding habits, and they can serve as sentinels for AMR in wildlife (7, 9–12). While studies on AMR in these animals are limited, particularly outside North America (13) and Europe (9, 10), wild boars, a common species studied in this context, have been shown to harbor a variety of AMR genes, especially for tetracyclines and aminoglycosides, according to recent research in Portugal (14).

Other studies focused on medium-sized scavenging/synanthropic Canidae like foxes and coyotes (15). A Norwegian study of the red fox (*Vulpes vulpes*) utilizing culture and PCR methods demonstrated a higher occurrence of extended-spectrum beta-lactamase (ESBL)-producing bacteria and related genes in foxes living in areas with a higher human population density (16). Using metagenomics, studies from the United States (13) and Mexico (17) found a variety of ARGs in coyotes (*Canis latrans*), a meso-carnivore canid and synanthropic species. Coyotes, along with other wildlife such as raccoons (*Procyon lotor*), Virginia opossums (*Didelphis virginiana*), and stray dogs, demonstrated a higher prevalence of ARGs related to aminoglycosides, beta-lactams, macrolides, quinolones, and tetracyclines compared to domestic dogs (13).

The Middle East region represents a further gap in wildlife AMR awareness (18). This is a critical data gap, as AMR in the Middle East is a rapidly emerging concern for human and veterinary health (19, 20). Among wildlife, data on synanthropes and scavenger species are also very limited. Therefore, we sought to assess the occurrence of AMR in golden jackals (GJs) living near humans in Israel, with a specific focus on beta-lactamase and quinolone resistance genes. The GJ (*Canis aureus*) is a common, medium-sized carnivore of the Canidae family (21, 22). The Israeli GJ population size has increased drastically countrywide during the last decade, following its spread into new geographical regions mostly in association with human settlements (23–25). The GJ is a synanthropic species, and its association with humans makes it a potential vehicle for many pathogens, especially zoonotic disease agents such as rabies (25).

Overall, there are numerous gaps in the literature with respect to AMR among wildlife. Particularly, most studies of scavenging/synanthropic species include a small number of animals or solely rely on culture-based methods. This study fills several such research gaps by applying culture-independent methods (both molecular and metagenomic sequencing) on a relatively large cohort of over 100 animals, to elucidate the resistome of a scavenging/synanthropic canine species. Furthermore, this study is one of few to assess AMR in wildlife in the Middle East region. By assessing the prevalence of select ARGs as well as the resistome of GJ, we present findings that broaden the understanding of AMR carriage in scavenger species in the region.

## MATERIALS AND METHODS

### Animal sampling and rabies and *Toxoplasma* tests

The study involved rectal swab samples obtained from GJ during predator and rabies control activities of the Israel Nature and Parks Authority (INPA) across four geographic regions. Animals were also tested for rabies oral vaccine uptake and *Toxoplasma*. The detailed sampling methodology was previously described in detail (25) and is also summarized in the supplemental methods.

### Real-time qPCR

DNA was extracted from ESwab fluid using the DNeasy PowerSoil Kit and quantified with a Qubit device. Five singleplex Taqman quantitative PCR (qPCR) assays were used to detect and quantify class 1 integron-integrase *Intl1*, three beta-lactamase genes (*bla*CTXM-15, *bla*SHV, *bla*TEM1), and *qnrS* (quinolone resistance). Gene abundance was normalized to the 16S *rRNA* gene. Quality control was achieved using custom plasmids and control strains for gene quantification. The primer and probe sequences are in Table S1. For further details on extraction and qPCR, see the supplemental methods.

### Metagenomic sequencing

We applied metagenomics on a subset of jackal samples from all study regions. The samples were chosen for this subset based on sample suitability in terms of DNA quality and quantity as well as the presence of more than two beta-lactamase genes during the qPCR screen.

Samples were sequenced on the Illumina NovaSeq6000 platform (2 × 150 sequencing) using the Illumina library preparation Nextera protocol. Twenty nanograms of each sample was processed using 1/5 of the reaction volumes described as per standard Illumina protocol. After fragmentation and cleanup, each sample was indexed in a 10-cycle PCR reaction.

### Gut microbiome analysis

Microbial community analysis was conducted using R (version 4.3.0) with the tidyverse, ggplot2, vegan, and microeco packages. Samples were normalized using the microeco package, and no OTUs were excluded to avoid biases from background contamination. Diversity was assessed using the Shannon diversity index and the Bray–Curtis dissimilarity index, and statistical significance was determined by permutational multivariate analysis of variance (PERMANOVA). The differential abundance of microbial communities and ARGs was analyzed using a random forest model with the microeco package. ARG normalization was performed with args_oap, and microbiome analysis was repeated with these normalized ARGs. A Procrustes analysis was also conducted to assess the correlation between the microbiome and normalized resistome at the family level. For more details, see the supplemental methods.

### Bioinformatics analysis

**Fig 1:**
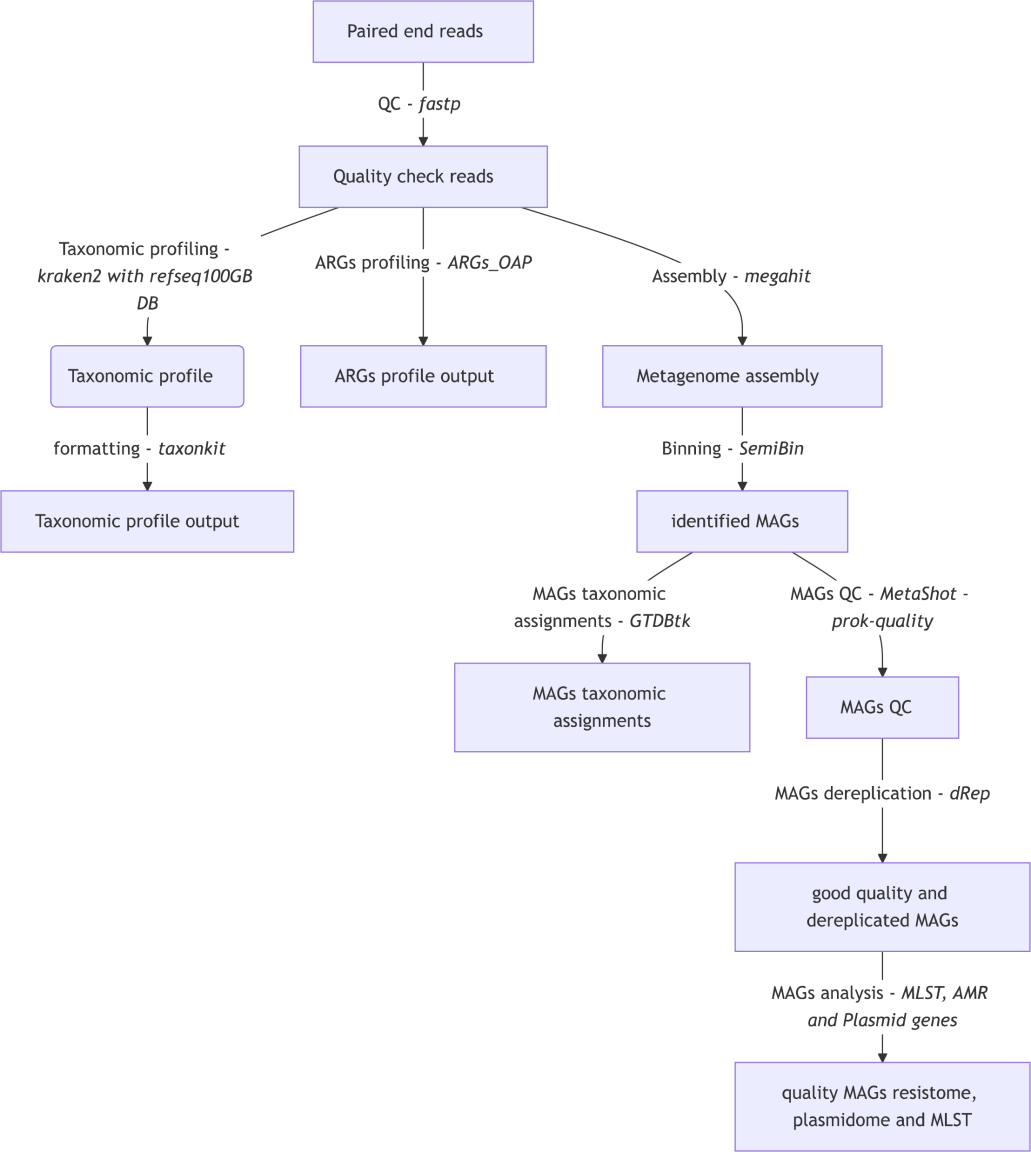
Bioinformatic analysis workflow.

The steps of the analysis are summarized in Fig. 1. Raw sequence reads for the selected samples underwent quality control and trimming with fastp (v.0.23.2) (26). A total of 18 samples were included for analysis (ranging between 3,599,930 and 51,533,717 read pairs, with a mean 37,086,710 read pairs). Taxonomic and ARGs profiling was performed on the reads following quality control. Following metagenome assembly, the metagenome-assembled genomes (MAGs) were isolated and underwent species identification, resistome, and mobilome proflling (see supplemental methods).

### Statistical analysis

Comparisons between specimen characteristics (region, age class, sex) and *Toxoplasma* and rabies vaccination test results and ARG prevalence and gene copies were made in R software (ver 4.1.3). We used χ^2^ and Fisher’s exact tests to compare ARG prevalence to specimen characteristics and Wilcoxon and Kruskal–Wallis tests to compare normalized ARG copy numbers to specimen characteristics. The comparisons were reported as mean ± standard deviation (SD) for the quantitative variables and percentage for the categorical variables. *P* < 0.05 was regarded as statistically significant. All *P* values are false discovery rate-adjusted.

## RESULTS

The sampling effort took place from August 2019 to April 2020, and during this time, 111 GJ specimens were collected. The distribution of specimens across regions, sex, and age estimation is summarized in Table S2.

### Rabies oral vaccination and *Toxoplasma* tests

A total 106 of 111 GJ were examined for rabies vaccination and *Toxoplasma*. Immunity to rabies virus was detected based on exposure to oral vaccine (tetracycline test) in 52.8% of the GJ. *Toxoplasma* antibodies were found in 29.2% of the GJ. For more information on these conditions and further analysis, the reader is referred to our GJ fecal microbiome study on the same cohort (25).

### Abundance and quantification of ARGs in the GJ via qPCR

**Fig 2:**
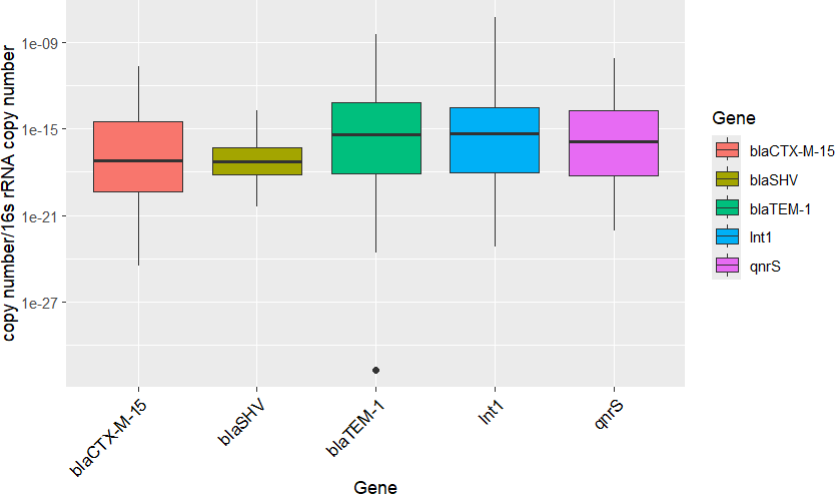
Copy number of *bla*CTX-M-15, *bla*SHV, *bla*TEM-1, *qnrS*, and *Int1* per µL, normalized by 16S *rRNA* gene copies.

Testing for ARGs via qPCR showed the notable presence of the selected ARGs in the GJ fecal samples (Table 1). Following adjustment to copy numbers, higher copy numbers of Int1, *bla*TEM-1, and *qnrS* than *bla*CTXM15 and *blaSHV* were observed (Fig. 2). Overall, 96.4% of jackals were positive for *blaTEM-1*, 94.6% were positive for Int1, 87.4% were positive for *qnrS*, 51.4% were positive for *blaCTX-M15*, and 15.3% were positive for *blaSHV*. There was no statistically significant association observed between geographic region and the presence of ARGs, except for the high prevalence (75%) of *bla*CTX-M15 in region group 4 compared to a relatively low prevalence of *bla*CTX-M15 (37%) in region group 1, which was borderline significant (*P* = 0.07). By ARG copy number (normalized by 16S *rRNA* gene copy number) (Table S3), no statistical significance was observed regarding the relationship between copy number of the *bla*TEM-1, Int1, *blaSHV*, *bla*CTXM15, and *qnrS* genes and region (*P* > 0.16). On age class comparison, only the presence of *blaTEM-1* was found to be significant (*P* = 0.04) as more adults were positive (100%) for *blaTEM-1* than juveniles (93.75%) and subadults (90.63%). By copy number, *bla*CTX-M15 relationship with age class was borderline significant (*P* = 0.07) as adults had higher copy number (1.05E^−12^ copy number/16S *rRNA* copy number, 2.27E^−23^–2.26E^−11^) for *bla*CTX-M15 than juveniles (1.23E^−15^ copy number/16S *rRNA* copy number, 2.95E^−25^–8.68E^−15^) and subadults (2.38E^−15^ copy number/16S *rRNA* copy number, 2.78E^−24^–2.86E^−14^).

**TABLE 1:** Percent prevalence of ARGs by jackal demographic factors

		*bla*CTX-M15	*bla*SHV	*bla*TEM-1	*qnr*S	*Int*l1
Sex	Male (*n* = 54)	51.8 ± 50	14.8 ± 36	96.3 ± 19	92.6 ± 26	94.4 ± 23
	Female (*n* = 57)	50.9 ± 50	15.8 ± 37	96.5 ± 19	82.4 ± 38	94.7 ± 23
Age class	Adult (*n* = 63)	52.4 ± 50	14.3 ± 35	100.0	87.3 ± 34	95.2 ± 21
	Subadult (*n* = 32)	50.0 ± 51	18.8 ± 40	90.6 ± 30	90.6 ± 30	93.8 ± 25
	Juvenile (*n* = 16)	50.0 ± 52	12.5 ± 34	93.8 ± 25	81.3 ± 40	93.8 ± 25
Region	1 (*n* = 40)	37.5 ± 49	12.5 ± 33	92.5 ± 27	90.0 ± 30	92.5 ± 27
	2 (*n* = 39)	53.8 ± 51	23.1 ± 43	100.0	87.2 ± 34	94.9 ± 22
	3 (*n* = 16)	56.2 ± 51	12.5 ± 34	93.8 ± 25	81.2 ± 40	100.0
	4 (*n* = 16)	75.0 ± 45	6.2 ± 25	100.0	87.5 ± 34	93.8 ± 25
Bone tetracycline	Negative (*n* = 50)	46 ± 50	20 ± 29	96 ± 19	88 ± 35	94 ± 23
	Positive (*n* = 56)	53.6 ± 50	8.9 ± 40	96.4 ± 20	85.7 ± 33	94.6 ± 24
*Toxoplasma* Ab	Negative (*n* = 75)	52 ± 50	8 ± 27	97.3 ± 16	86.7 ± 34	94.7 ± 23
	Positive (*n* = 31)	45.1 ± 51	25.8 ± 44	93.5 ± 25	87 ± 34	93.5 ± 25
Total	*n* = 111	51.4 ± 50.2	15.3 ± 36.2	96.4 ± 18.7	87.4 ± 33.4	94.6 ± 22.7

**Fig 3:**
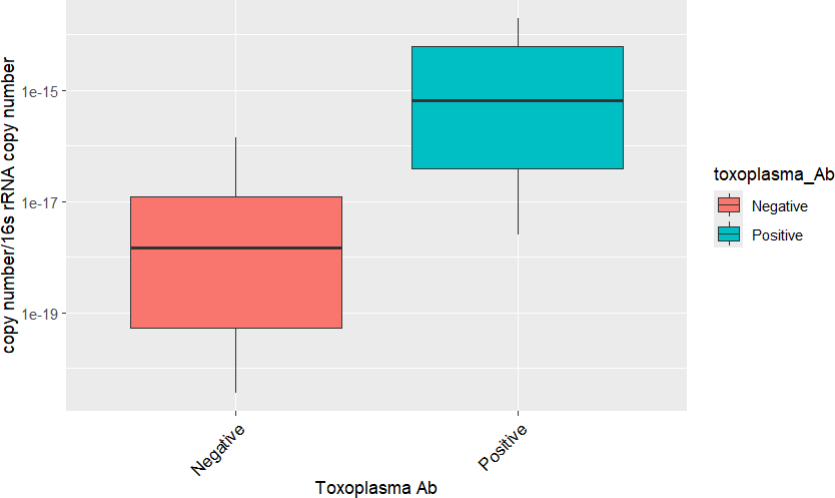
Copy number of *bla*SHV in *Toxoplasma*-seropositive and *Toxoplasma*-seronegative animals for *Toxoplasma*, normalized by 16S *rRNA* gene copies.

Sex was not found to have a significant association with ARG presence and ARG copy number of all genes (*P* > 0.15 for all genes). The association between *bla*SHV and exposure to *Toxoplasma* was significant (*P* = 0.02) (seropositive jackals were more likely to be positive (25.8%) for *bla*SHV than seronegative jackals that were (8%). *Toxoplasma*-seropositive specimens (Fig. 3) were found to have significantly higher copy numbers (5.29E^−15^ copy number/16S *rRNA* copy number, 2.53E^−18^–1.95E^−14^) for the *blaSHV* gene (*P* = 0.02) than *Toxoplasma*-negative ones (1.87E^−17^ copy number/16S *rRNA* copy number, 3.62E^−21^–1.41E^−16^). The rabies oral vaccine bioindicator, tetracycline in bone, was not found significant regarding the presence of all genes (*P* > 0.16). By copy number, the relationship between tetracycline in bone and *qnrS* was approaching significance (*P* = 0.06); positive specimens were found with higher copy number (3.23E^−12^ copy number/16S *rRNA* copy number, 1.09E^−21^–7.71E^−11^) for *qnrS* than negative (1.46E^−12^ copy number/16S *rRNA* copy number, 7.85E^−23^–5.76E^−11^) specimens.

### Gut microbiome analysis

**Fig 4:**
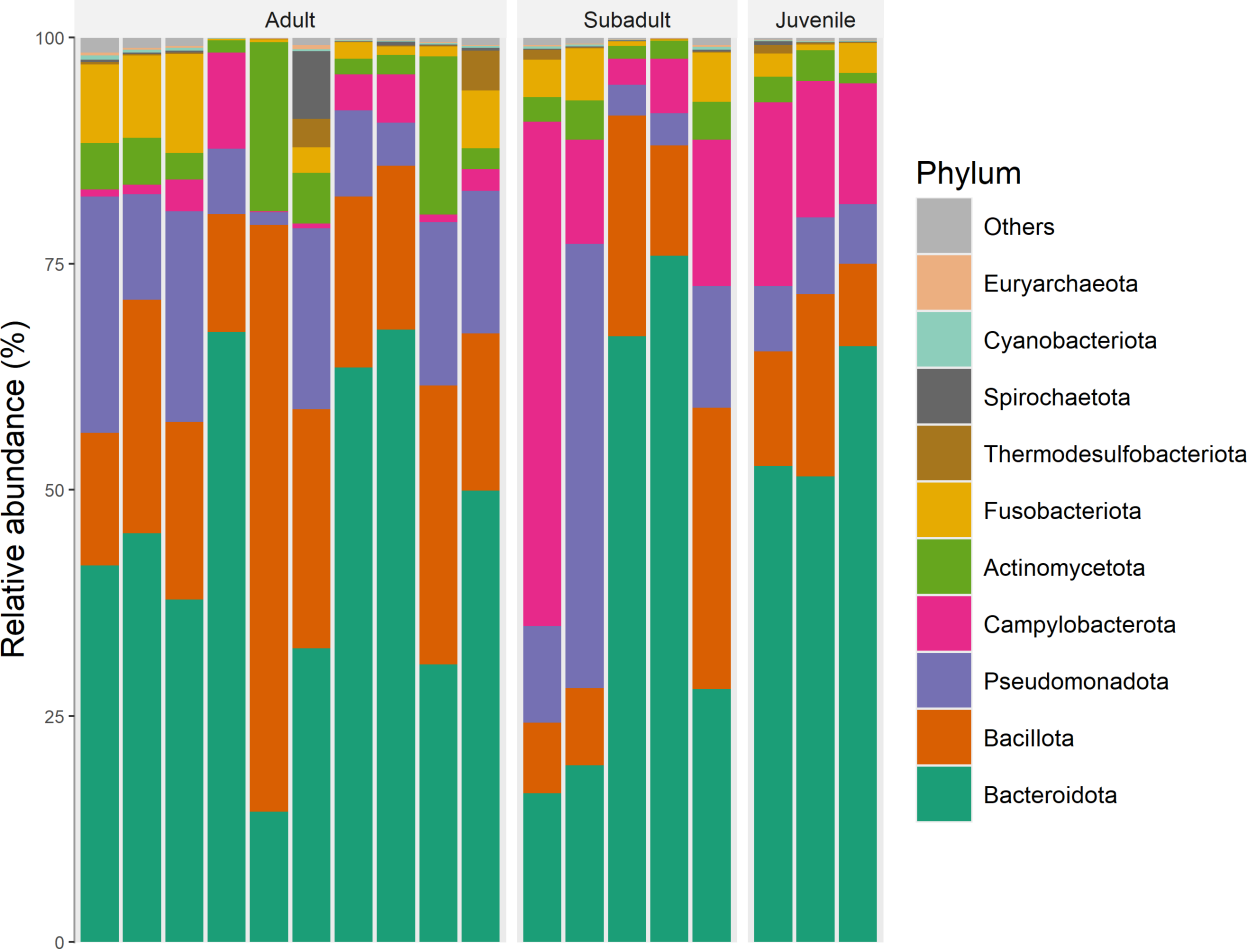
Relative abundance of top 10 most abundant bacterial phyla found among studied GJ samples, clustered by age class.

From the total 111 GJ fecal samples, we selected 18 samples for metagenomic sequencing. Bacterial taxa varied largely in their proportions between individuals; 49 phyla were found across all samples, but only 10 phyla had abundance >0.1% and accounted for more than 99% of relative abundance (Fig. 4; Table S4). The most abundant bacterial phyla were the Bacteroidota (46% ± 19.5%), Bacillota (20.9% ± 13.1%), and Pseudomonadota (13.3% ± 11.4%), where abundance is noted as mean ± SD. Among them, a total of 701 bacterial families were identified, but only 53 had abundance >0.1%. These families accounted for 95.2% of total abundance (Table 2). The most abundant families were *Bacteroidaceae* (34.2% ± 15.6%), *Lachnospiraceae* (9.7% ± 10.3%), and *Helicobacteraceae* (8.9% ± 12.9%).

**TABLE 2:** Relative abundance of leading bacterial families

Family	Mean (%)	Min (%)	Max (%)	Median (%)	Iqr (%)
*Bacteroidaceae*	34.2	11.6	62.5	31.1	20.6
*Lachnospiraceae*	9.7	2.3	44.9	6.4	9.8
*Helicobacteraceae*	8.9	0.01	54.3	4.5	*11.4*
*Prevotellaceae*	7.7	0.3	47.0	1.5	2.7
*Succinivibrionaceae*	5.7	0.1	44.9	1.8	5.1
*Clostridiaceae*	4.4	0.5	18.1	2.9	4.0
*Fusobacteriaceae*	3.5	0.1	10.7	0.9	4.8
*Enterobacteriaceae*	2.9	0.1	13.1	1.0	2.7
*Coriobacteriaceae*	2.8	0.3	17.8	1.0	1.4
*Oscillospiraceae*	1.3	0.2	3.1	0.1	1.2
*Selenomonadaceae*	1.2	0.03	13.1	0.8	0.5
*Tannerellaceae*	1.2	0.1	3.2	0.8	1.8
*Sutterellaceae*	1.1	0.1	5.4	0.7	1.1

Analysis of bacterial genera revealed 3,674 genera, of which 60 had abundance >0.1% and accounted for 89.12% of total abundance (Table 3). The most abundant genera in the GJ were *Phocaeicola* (26.5% ± 15.9%), *Helicobacter* (8.9% ± 12.9%), *Bacteroides* (7.4% ± 4.8%), and *Segatella* (6.5% ± 13.6%).

**TABLE 3:** Relative abundance of leading bacterial genera

Genus	Mean (%)	Min (%)	Max (%)	Median (%)	Iqr (%)
*Phocaeicola*	26.5	4.6	58.0	22.2	22.3
*Helicobacter*	8.9	0.01	54.3	4.5	11.4
*Bacteroides*	7.4	1.2	16.5	6.4	6.0
*Segatella*	6.5	0.04	46.3	0.3	0.9
*Anaerobiospirillum*	5.7	0.04	44.8	1.8	5.1
*Mediterraeibacter*	5.4	0.3	35.2	3.4	4.6
*Clostridium*	3.8	0.4	17.5	2.3	2.2
*Fusobacterium*	3.1	0.1	9.4	1.0	4.4
*Collinsella*	2.8	0.3	17.8	0.8	1.4
*Blautia*	2.0	0.2	8.6	0.3	2.1
*Escherichia*	1.8	0.02	11.9	0.1	0.6
*Megamonas*	1.2	0.01	13.0	0.7	0.5
*Parabacteroides*	1.2	0.1	3.1	0.7	1.8
*Sutterella*	1.1	0.1	5.4	0.01	1.1

#### Alpha diversity analysis

**Fig 5:**
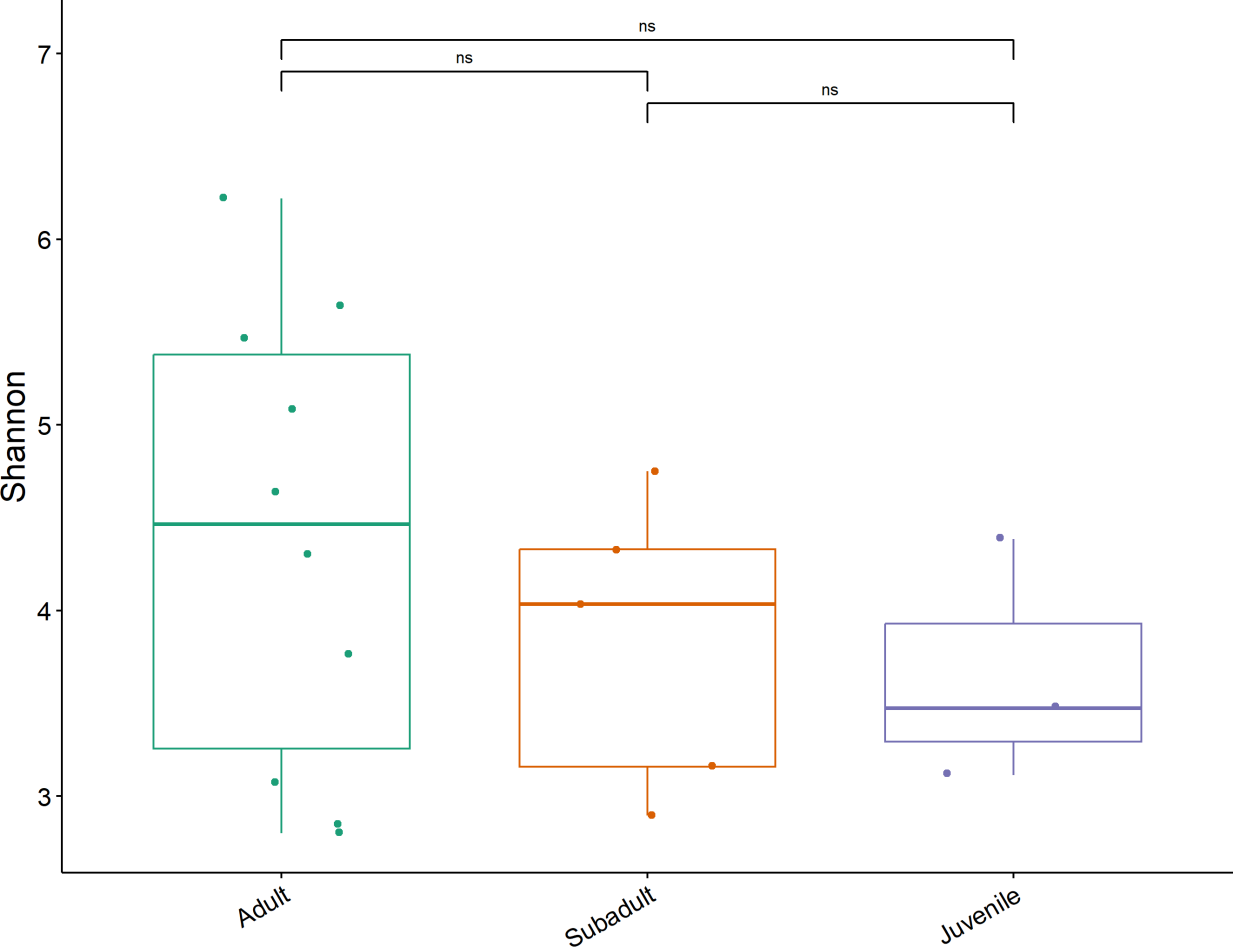
Alpha diversity by Shannon index between age classes. Significance was determined with the Wilcoxon rank sum test. ns = no significance.

Alpha diversity was measured using the Shannon diversity index. The alpha diversity was not significantly different by age class (Fig. 5); region; sex; *Toxoplasma* sero-status; *bla*CTX-M15, *bla*SHV, and *bla*TEM-1 positivity in qPCR; and bone tetracycline using the Wilcoxon rank sum test (possibly due to small sample size). We did, however, observe a trend toward higher Shannon diversity in adults, females, *bla*SHV positivity, and bone tetracycline.

#### Beta-diversity analysis

**Fig 6:**
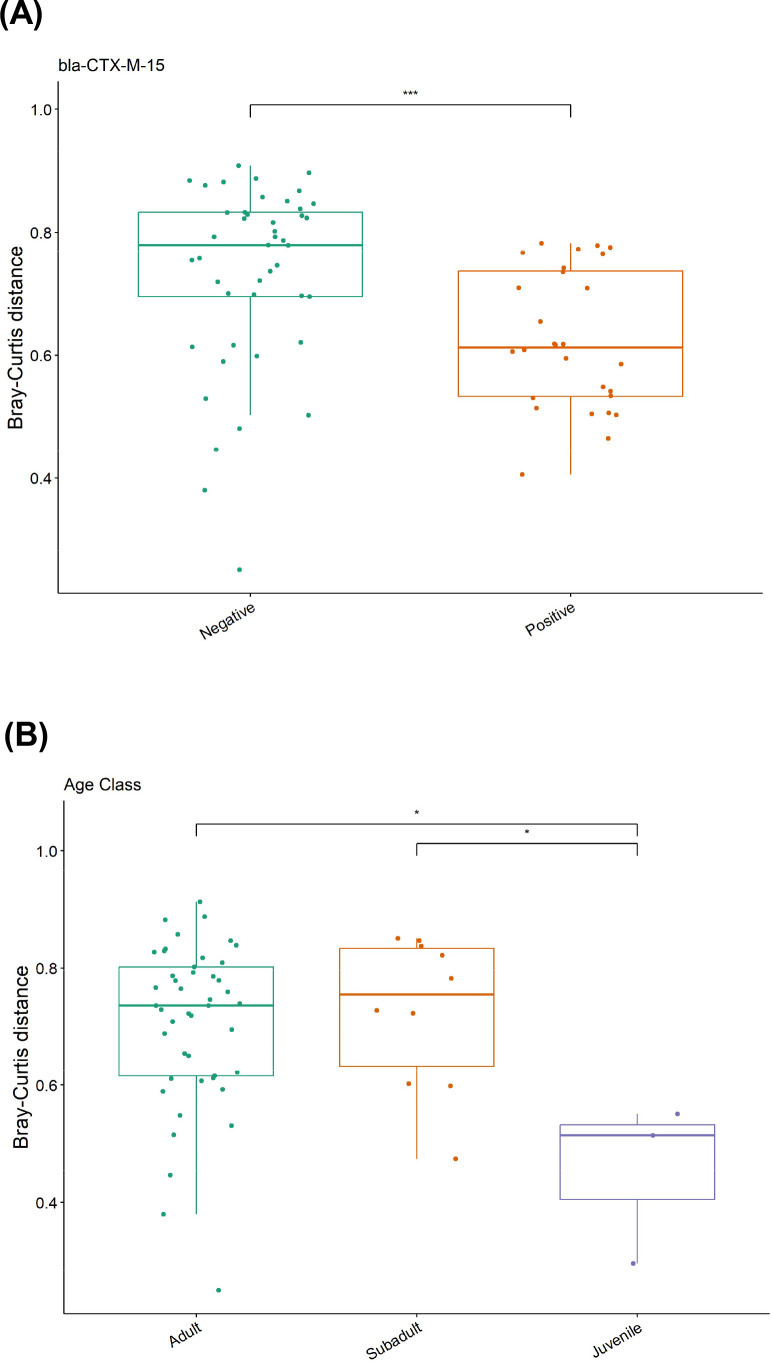
(**A**) Beta-diversity in Bray–Curtis dissimilarity index between *bla*CTX-M15-negative and *bla*CTX-M15-positive. Significance was determined with the Wilcoxon rank sum test. ***P < 0.0001 (**B**) Beta-diversity in Bray–Curtis dissimilarity index by age class. Significance was determined with the Wilcoxon rank sum test. *P < 0.05, ns = no significance.

Beta-diversity was compared between groups using the Bray–Curtis dissimilarity index. A significant difference was observed between *bla*CTXM15-positive and *bla*CTXM15-negative GJs (*F* = 1.19, *P* = 0.0002, Wilcoxon rank sum) and age class (*F* = 1.44, *P* = 0.0420 subadult-juvenile; *F* = 1.68, *P* = 0.0232 juvenile-adult, Wilcoxon rank sum, *F* = 0.72, *P* = 0.6594 subadult-adult) (Fig. 6). No significance was observed for region, sex, *Toxoplasma* sero-status, *bla*SHV, blaTEM-1, or bone tetracycline.

Despite a significant difference in Bray–Curtis index, dissimilarity could not be demonstrated between microbial communities based on *bla*CTXM15 status using PERMANOVA (*P* = 0.278, 999 permutations). Dissimilarity was demonstrated only between juvenile and adult jackal samples using PERMANOVA (*P* = 0.070, 999 permutations) (Fig. S2A). We then compared groups using non-metric multidimensional scaling and observed no obvious difference between the groups (*bla*CTXM positivity and age class), but the differing ellipses may indicate a difference in variances (Fig. S2B).

#### Differential abundance

**Fig 7:**
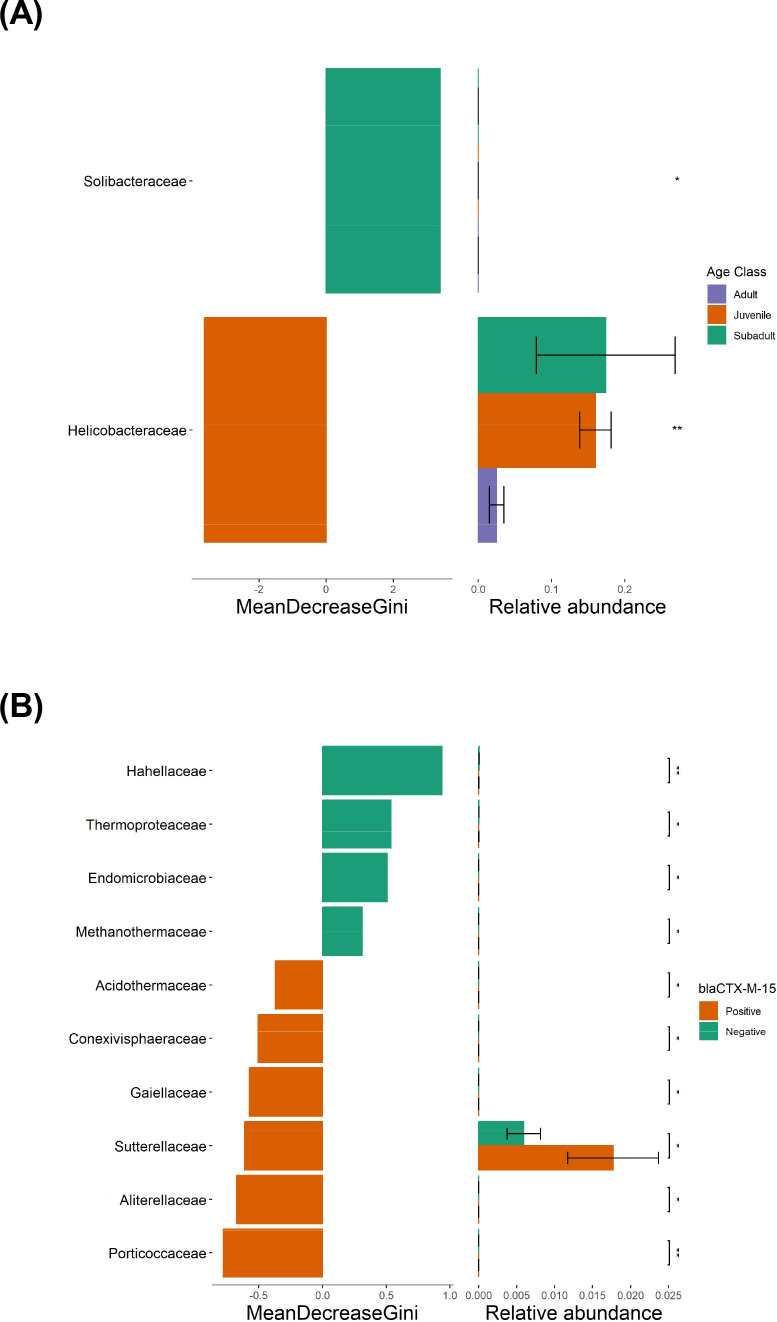
(**A**) Random forest analysis identified *Helicobacteraceae* as differentially abundant across age classes. *P < 0.05, **P < 0.01. (**B**) Random forest identified *Sutterellaceae* as differentially abundant across *bla*CTXM15 presence.

Differential abundance of bacterial taxa was determined by random forest with 999 permutations to identify community differences across groups. *Helicobacteraceae* was differentially abundant across age classes (the relative abundance of *Helicobacteraceae* was greater in juvenile and subadult GJs compared to adult GJs), and *Sutterellaceae* was differentially abundant across *bla*CTX-M15 positive and *bla*CTX-M15-negative samples (relative abundance in *Sutterellaceae* was greater in *bla*CTX-M15-positive GJs compared to *bla*CTX-M15-negative GJs) (Fig. 7).

#### Alpha diversity analysis for normalized resistome

Alpha diversity of normalized resistome features was measured using the Shannon index. Age, region, sex, *Toxoplasma* status, *bla*CTXM15, *bla*SHV, *bla*TEM-1, and tetracycline bone were all not found to be significantly associated with resistome alpha diversity using the Wilcoxon rank sum test (Fig. S3).

#### Beta-diversity analysis of normalized resistome

**Fig 8:**
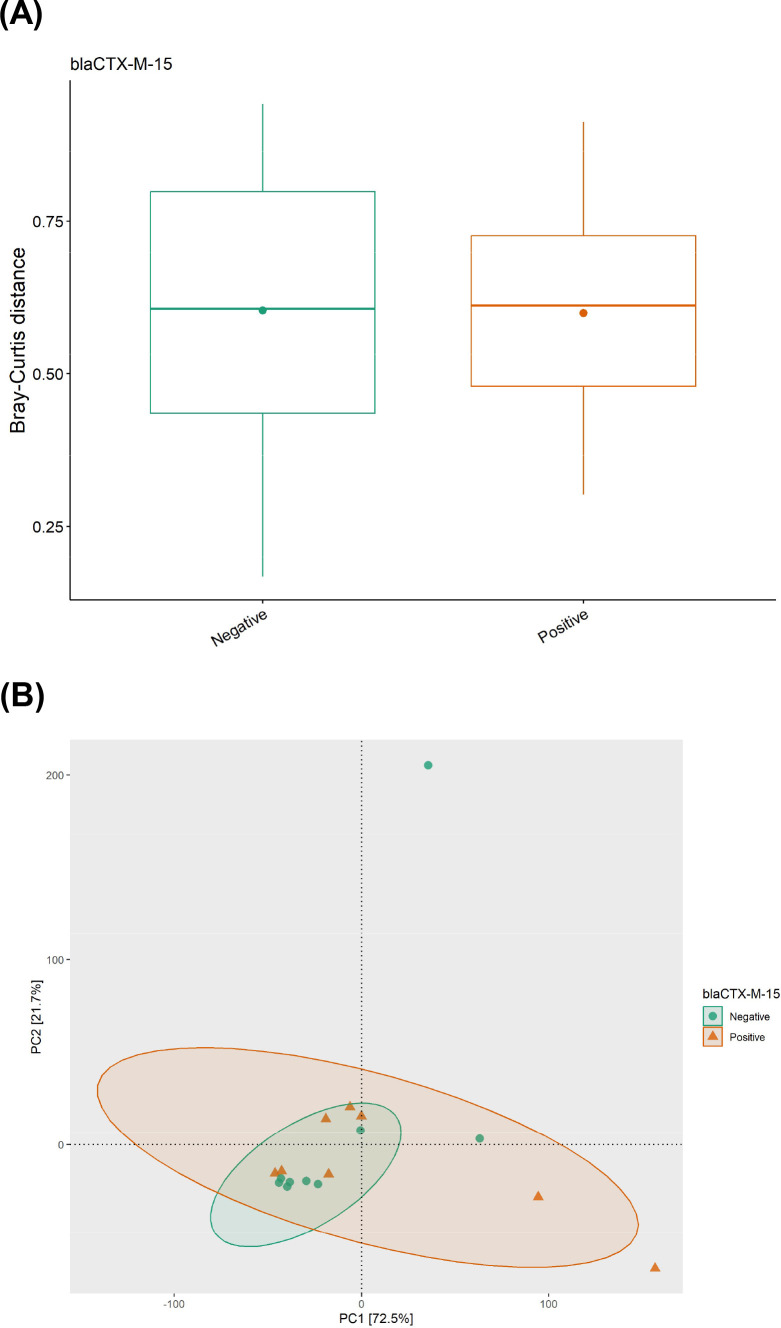
(**A**) Beta-diversity in Bray–Curtis dissimilarity index based on *bla*CTX-M status by qPCR screen in GJs. (**B**) Principal component analysis plot demonstrates no discernible difference between *bla*CTXM status and ARG presence.

Beta-diversity was compared between groups using the Bray–Curtis dissimilarity index. No significant difference was observed when comparing age, region, sex, *Toxoplasma* status, *bla*CTXM15 (Fig. 8), *bla*SHV, *bla*TEM, or tetracycline bone using both Wilcoxon rank sum test and PERMANOVA.

#### Taxonomic profile and resistome profile correlation

**Fig 9:**
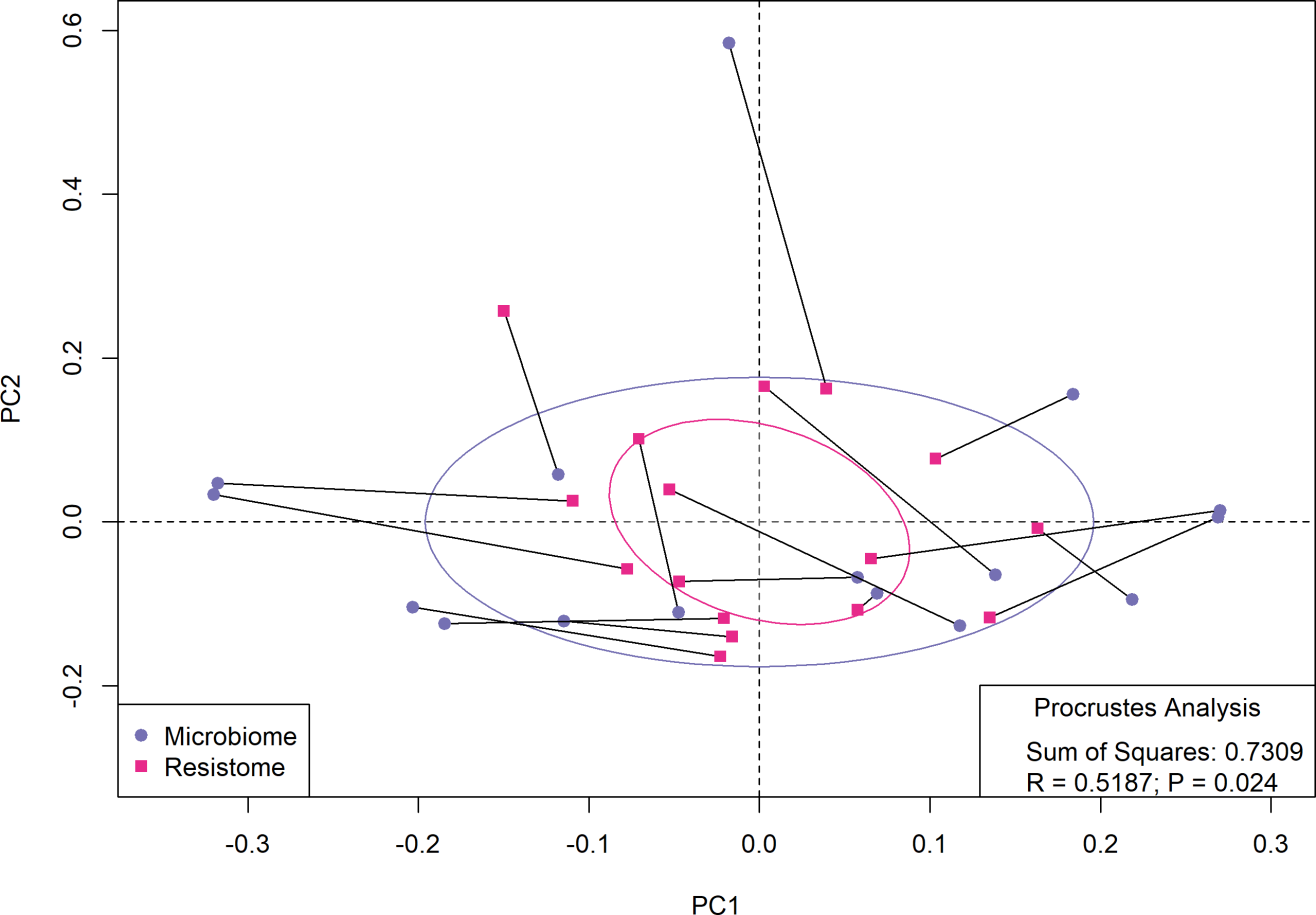
Procrustes analysis demonstrates a significant correlation between GJ resistome and community composition.

To evaluate the structural correlation between the microbiome and the normalized resistome, we performed a Procrustes analysis (Fig. 9). We found a moderate (*R* = 0.5187) and significant (*P* = 0.024) correlation between bacterial families and normalized ARGs data. This correlation suggests that shifts in either structure may influence the other.

### ARG normalization

**Fig 10:**
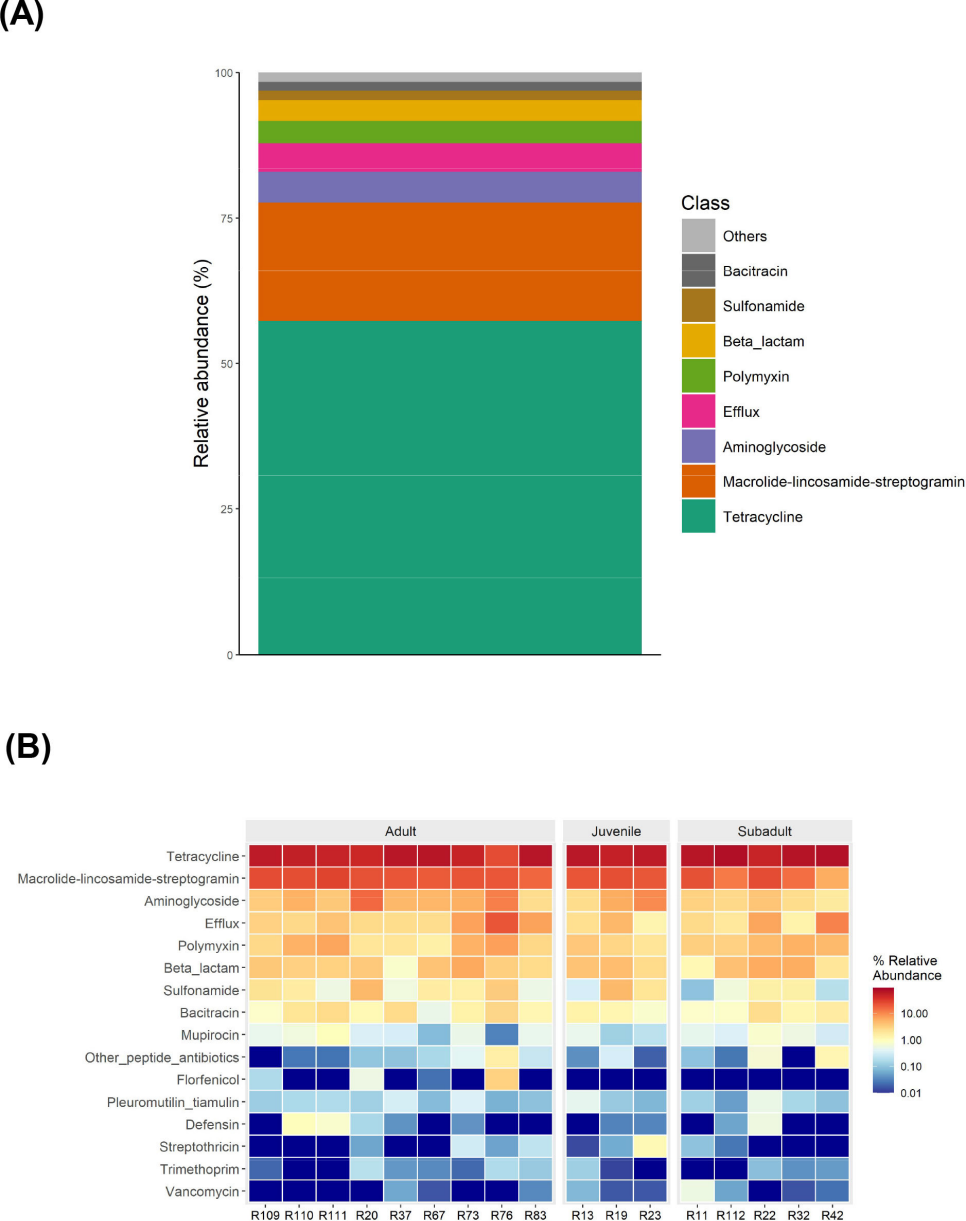
**(A**) Relative abundance of normalized ARGs by drug class. (**B**) Heat map of the abundance of all ARGs by antimicrobial class and age class.

We normalized ARGs by the number of 16S *rRNA* gene copies and identified 431 ARGs genes between all samples (Table S5) representing 24 antimicrobial classes (Fig. 10A). In all of the GJs, the tetracycline resistance genes were the most dominant (57.3% of all ARGs). Other dominant antimicrobial classes were macrolide-lincosamide-streptogramin (20.3%), aminoglycoside (5.3%), efflux (5%), polymyxin (3.8%), and beta-lactams (3.6%). A heat map of the relative abundance of all ARGs by antimicrobial class and age class is demonstrated in Fig. 10B.

The concordance between detected ARGs of interest using qPCR and metagenomics, respectively, was 96.4% vs 64.7% for *bla*TEM, 15.3% vs 11.7% for *bla*SHV, 51.4% vs 5.9% for *bla*CTX-M15, and 87.4% vs 17.6% for *qnr*S. Individual sample data are provided in Table S6.

### Metagenome-assembled genomes

MAG identification using SemiBin (with dog gut binning method) isolated a total of 1195 MAGs, of which 307 MAGs were of high-quality according to the MIMAG criteria (completeness > 90%, contamination < 5%, and identification of *rRNA* and tRNA genes). Using Abricate with the Comprehensive Antibiotic Resistance Database (CARD database), 98 high-quality MAGs carrying ARGs were identified, with eight clinically relevant taxa harboring four beta-lactamase, three tetracycline, one streptothricin, and one glycopeptide resistance genes; AMRFinderPlus analysis of 43 high-quality MAGs revealed six relevant taxa with four beta-lactamase, three tetracycline, and one streptothricin resistance genes. Multilocus sequence typing (MLST) recovered six profies for eight MAGs (Table S7), but only one MAG, namely, R83_env.dog_gut_bin.59 (a *Campylobacter coli*), had a full ST (sequence type) assignment (ST1770). Using Abricate with the plasmidfinder database, eight high-quality MAGs were found carrying seven plasmid-associated genes, with one MAG (R83_env.dog_gut_bin.59, *C. coli*) also carrying ARGs (*bla*OXA-193, *bla*OXA-605, and satA). Table 4 shows the 17 MAGs related to clinically relevant taxa identified using Abricate-CARD and AMRFinderPlus, including seven genera from four phyla. Most of the MAGs hold tetracycline ARGs (12 MAGs) and fewer beta-lactam ARGs (5 MAGs), one MAG included streptothricin ARG, and one MAG included vancomycin ARG.

**TABLE 4:** Important MAGs identified using Abricate-CARD and AMRFinderPlus^*a*^

MAG Unique_ID	Taxon	Phylum	Plasmid	ARGs
R19_env.dog_gut_bin.64*^#^	*E. coli*	Proteobacteria	–	*bla*EC*, EC-18^#^
R76_env.dog_gut_bin.13*^#^	*E. coli*	Proteobacteria	–	*bla*EC*, AmpC beta-lactamase^#^
R83_env.dog_gut_bin.59*^#^	*C. coli*	Campylobacterota	repUS53_1_repB(pJB01)	*bla*OXA-193*, OXA-605^#^, satA*
R110_env.dog_gut_bin.2*^#^	*Clostridium perfringens*	Firmicutes	–	tetA(*P*) ^#^*, tetB(*P*) ^#^
R111_env.dog_gut_bin.3*^#^	*Clostridium perfringens*	Firmicutes	–	tetA(*P*) ^#^*
R37_env.dog_gut_bin.54^#^	*Prevotella*	Bacteroidota	–	tet (37) ^#^
R37_env.dog_gut_bin.25*^#^	*Prevotella copri*	Bacteroidota	–	cfxA6^#^*, tet (37) ^#^
R32_env.dog_gut_bin.26^#^	*Prevotella copri*	Bacteroidota	–	tet (37) ^#^
R73_env.dog_gut_bin.11^#^	*Prevotella copri*	Bacteroidota	–	tet (37) ^#^
R20_env.dog_gut_bin.135^#^	*Prevotella* sp015074785	Bacteroidota	–	tet (37) ^#^
R22_env.dog_gut_bin.31^#^	*Prevotella* sp015074785	Bacteroidota	–	tet (37) ^#^
R32_env.dog_gut_bin.21^#^	*Prevotella* sp900552675	Bacteroidota	–	tet (37) ^#^
R111_env.dog_gut_bin.29*	*Anaerobiospirillum succiniciproducens*	Proteobacteria	–	tet(G)*
R13_env.dog_gut_bin.54*	*Anaerobiospirillum succiniciproducens*	Proteobacteria	–	tet(G)*
R20_env.dog_gut_bin.76*	*Anaerobiospirillum succiniciproducens*	Proteobacteria	–	tet(G)*
R67_env.dog_gut_bin.18*	*Anaerobiospirillum succiniciproducens*	Proteobacteria	–	tet(G)*
R32_env.dog_gut_bin.5*	*Anaerobiospirillum*	Proteobacteria	–	*bla*TEM*
R37_env.dog_gut_bin.6^#^	*Erysipelatoclostridium ramosum*	Firmicutes	–	vanR_gene_in_vanG_cluster^#^

^*a*^ #, found in Abricate-CARD; *, found in AMRFinderPlus.

## DISCUSSION

### Study summary

Our study revealed a high prevalence and notable diversity of ARGs in GJs, with tetracycline resistance genes being the most prevalent. By means of qPCR, *bla*TEM-1, *Int*1, and *qnr*S were the most frequently identified ARGs, with their prevalence consistent across regions and sexes. Adults had higher *bla*TEM-1 positivity, and *Toxoplasma*-seropositive jackals showed increased blaSHV presence, suggesting microbiome differences linked to infection. Metagenomic analysis identified over 20 ARG classes, predominantly related to tetracyclines, macrolides, and beta-lactams but no carbapenemases. The main clinically relevant ARGs found using metagenomic analysis were *tet*(a), *tet(*O) (tetracycline), *erm*(B) (macrolide), and *sul*1 and *sul*2 (sulfonamide). These findings highlight the GJs as important AMR reservoirs in human-impacted environments, particularly near agricultural and livestock settings.

### Comparison with other Canidae studies

Our study revealed a high prevalence and diversity of ARGs in the GJ in northern Israel using qPCR and shotgun metagenomic approaches. Upon broadly screening 111 jackals using qPCR, we found the highest prevalence in the beta-lactamase gene (*blaTEM-1*), the integron gene (*Int1*), and the quinolone resistance gene (*qnrS)*. This prevalence was consistent and independent of sampling region and sex. In a study of red foxes from Portugal using qPCR, a higher abundance of ARGs was found in an area with greater human impact (27). Given that the GJ is a synanthropic species that thrives near human settlements, relying heavily on food sources associated with human activities and livestock production and considering the substantial human impact across all sampling regions in our study, we believe that this differential effect was not found here (21). We observed that *bla*TEM-1-positive specimens were more frequent (*P* = 0.04) in adult GJs compared to younger ones. Similar observations regarding age have not been reported in other studies. Therefore, we believe this finding may be related to increased exposure in adult GJs compared to juveniles, although further corroboration is required.

Regarding AMR and ARGs, similar studies in Canidae species are scarce and are often culture-based (15). One case study of Italian GJ using PCR methods observed *bla*TEM-1 and group of *tet* genes from GJ intestine samples (28). In the Iberian wolf (*Canis lupus signatus*), only 5.5% ESBL-producing *Escherichia coli* isolates were recovered from samples and found to harbor blaTEM52, *bla*SHV12, blaCTXM1, and *bla*CTXM14 (29). In this study, we tested similar ESBL genes (*blaCTXM15*, *blaTEM-1*, and *blaSHV*) and found a much higher prevalence (15.3%–96.4%). Those findings can be associated with the Iberian wolf acting as a wild predator and traveling large distances for its wild prey compared to the GJ that relies on agriculture and human products, species-specific microbiota, local epidemiology, or due to differences in gene prevalence in bacterial isolates vs metagenomic DNA samples. Red foxes (*V. vulpes*) from Portugal had a lower prevalence of select genes, when studied using qPCR, than GJs, that is, 12% were observed to be carrying *bla*TEM vs 96.4% of GJs and 2% observed to be carrying a quinolone resistance genes (*qnr*B) vs 87.4% of GJs (27). Although the red fox can act as a synanthropes species, the marked difference from the GJ derives from the presumably more natural diet of Portugal’s red fox and perhaps better sanitation around the red fox habitat.

### Comparison with wild boar

In another synanthropes species, the wild boar (*Sus scrofa*), a Portuguese study using high-throughput qPCR (14) demonstrated much lower prevalence of 11% ESBL genes (i.e., *cfx*A, *bla*SFO, and *bla*TEM) and 3% of quinolone resistance genes (*qep*A and *qnr*B4). Although both studies demonstrate a much lower prevalence of ARGs than the GJ, this may be due to microbiota differences and study location, and further comparative studies of different types of scavenger/synanthropic species are needed. The GJ and the wild boar typically share the same habitat and similar diet in our study area. Our findings demonstrate the unique situation of the GJ as an AMR sentinel, suggesting multiple sources of AMR transmission through human industries and livestock.

### *Toxoplasma* positivity and ARG prevalence

Another interesting finding is that *Toxoplsama*-seropositive GJ specimens had a higher percent positivity of the *bla*SHV gene and had higher *bla*SHV gene quantity than *Toxoplasma*-negative GJs. In a recent study (25) exploring the fecal microbiota of this cohort, we found that *Toxoplasma*-seropositive specimens had different beta-diversity compared to seronegative specimens. We suggest those differences may be associated with susceptibility to *Toxoplasma* or may be associated with the infection. This change in microbiota may also affect the risk of colonization with *bla*SHV-carrying bacteria. Limited studies in laboratory rats infected with *Toxoplasma* (30) noted marked changes in microbiome diversity from acute to chronic infection stages. That may support our findings in the GJ. Nevertheless, as our study lacked longitudinal sampling, this warrants further investigation.

### Metagenomic analysis

Our findings of the metagenomic analysis of a subset of GJ samples revealed a large amount of AMR genes belonging to more than 20 antibiotic classes. The majority of the genes were related to tetracycline and, in descending order, macrolides, aminoglycosides, efflux, polymyxin, beta-lactams, and sulfonamides. Clinically relevant genes that were found were *tet(a), tet(O)* from the tetracycline group, *erm(B)* in the macrolide group, sul1 and sul2 in the sulfonamide group, and also vanA from the vancomycin group found in one GJ specimen. No carbapenemases were found.

In a study of Chilean andean foxes (*Lycalopex culpaeus)* (31) using qPCR method, the tetracyclines and macrolides ARGs were also the most abundant.

In the coyote (*C. latrans*) study in North America, 27 ARGs from nine antibiotic classes were observed using shotgun metagenomics, and the majority of the ARGs were related to aminglycoside, beta-lactams, quinolone, and tetracycline resistance (13). Further comparison between the coyote to the GJ is needed, including larger studies of coyotes, but our results suggest a higher ARG prevalence in the GJ.

Our analysis revealed that tetracycline resistance genes were the most prevalent among the ARGs identified in the GJ samples (57.3%). This finding is concerning because tetracycline genes are classified as critically important antibiotics by the World Health Organization due to their broad-spectrum activity and effectiveness against various bacterial infections (32).

Tetracyclines, widely used in livestock farming and agriculture, are prevalent in nearly 90% of food-producing animals and are associated with high levels of antibiotic residues in animal excreta (33, 34). Their stability and slow degradation in the environment contribute to their persistence and the spread of tetracycline resistance genes (35). The high abundance of these genes in GJ samples from areas near livestock farms, agricultural settings, and landfills suggests that these environments could act as reservoirs for AMR dissemination in wildlife (36). Manure, rich in unmetabolized tetracyclines, is often used as fertilizer, leading to detectable levels of antibiotics in soils (33). The presence of antibiotics in soils contributes to environmental risk and the potential for ARG transfer in wildlife populations, such as GJ, that inhabit these environments.

Metagenomic analysis of the GJ cohort revealed similar phyla composition of the microbiome based on 16S-*rRNA* analysis that we performed on the full cohort (25). The results suggest the GJ microbiome is mainly comprised of Bacteroidota, Fusobacteriota, Firmicutes, Proteobacteria, and Campilobacterota. In the MAGs analysis, we detected 98 bins containing ARGs using Abricate-CARD or AMR-finder-plus. In both methods, we identified only six to eight bins with public health relevance which belong to the Proteobacteria, Firmicutes, Campylobacterota, and Bacteroidota phyla. Similar to ARG distribution described before, the majority of MAGs contained tetracycline resistance genes, beta-lactamases, and streptothricin- and vancomycin-related ARGs. In one sample, *C. coli* was found with a plasmid-borne gene, but ARG bearing plasmids were not found in other MAGs, possibly due to their absence or analytical limitations. The associated plasmid, *pJB01*, was recovered also in *Campylobacter jejuni* isolated from food animal production (37). Overall, most MAGs identified from the GJ did not harbor clinically important genes. Most of the bacterial MAGs from the GJ were not considered virulent or dangerous to humans, similar to findings in studies of urban bacteria (38). However, the limited number of ARGs identified in the MAGs could also be attributed to the limitations of the databases used, similar to observations in a study on domestic pigs (39).

### Study limitations

Our study has several limitations, primarily relating to the sampling methodology and, in the case of metagenomics, sample size and sequencing depth. We focused on wild GJs and collected specimens primarily through culling methods. This approach allowed us to obtain fresh samples and blood for further diagnostic tests, but sample collection was laborious and dependent on pre-scheduled culling efforts, thus limiting cohort size. A small subset of samples was selected for metagenomics due to limitations in quantity and quality of DNA extracts available for sequencing. We also observed discrepancies between our qPCR findings and the metagenomics, with a lower abundance of the genes identified in the metagenomes compared to qPCR results. We speculate that these discrepancies may be due reflect to the higher sensitivity of real-time PCR that may be associated with amplification, as shown in previous studies (40).

### Conclusions and future research

This study highlighted the abundance of clinically important ARGs among GJs and identifies the potential role of GJs as potential reservoirs and vehicles for ARGs. Understanding the factors driving the high prevalence of ARGs in these animals is crucial for developing strategies to mitigate the spread of AMR. Given the growing public health threat posed by AMR, further investigation into the role of wildlife as reservoirs and related transmission routes is crucial. Future research should employ a multifaceted approach, incorporating traditional antimicrobial susceptibility testing, isolate culturing, and culture-independent methods (i.e., metagenomic and qPCR). Further studies are needed to encompass a broader wildlife species and environments to better understand the unique ecological factors shaping AMR patterns. Additionally, jackals are a species readily capable of transboundary movements, which is a critical consideration in a region where countries are small and borders are close. A “One Health” approach is thus essential, requiring transboundary cooperation across the Middle Eastern region in order to track the spread of resistant bacteria through migratory wildlife populations and develop regional strategies to mitigate AMR global threats.

## Data Availability

Metagenomic DNA samples were deposited at Sequence Read Archive under the bioproject number PRJEB79102.

## SUPPLEMENTAL MATERIAL

Supplemental material msphere.00819-24-s0001.docx Supplemental text, figures, and tables.

ASM does not own the copyrights to Supplemental Material that may be linked to, or accessed through, an article. The authors have granted ASM a non-exclusive, world-wide license to publish the Supplemental Material files. Please contact the corresponding author directly for reuse.
