# Supplementary material for: Abundance of clinically relevant antimicrobial resistance genes in the golden jackal (Canis aureus) gut
Source: mSphere. 2025 Feb 13;10(3):e00819-24. doi: 10.1128/msphere.00819-24 (PMC11934335; doi:10.1128/msphere.00819-24)
Supplement: Supplemental material — Supplemental text, figures, and tables. [file msphere.00819-24-s0001.docx]

Abundance of Clinically-Relevant Antimicrobial Resistance Genes in the Golden Jackal (*Canis aureus*) Gut

**Supplementary materials**

**Table of Contents**

1. Supplementary methods

- Animal sampling
- Rabies and Toxoplasma tests
- Real-Time quantitative PCR (qPCR)
- Gut microbiome analysis
- Bioinformatics analyses

1. Supplementary Figures

- Figure S1. Distribution of GJ sampling regions on a map of center-north of Israel
- Figure S2. NMDS plots demonstrating differences between blaCTXM15 status and age classes
- Figure S3. Alpha diversity by Shannon index between age classes

1. Supplementary Tables

- Table S1. Primers and probes used in this study
- Table S2. General information of the studied GJ cohort
- Table S3. qPCR gene copy number by demographic variables
- Table S4. Relative Abundance of Leading Phyla
- Table S5. ARGs prevalence in GJ - metagenomics
- Table S6. Concordance between detection of ARGs with qPCR and metagenomic methods
- Table S7. Taxa recovered by MLST

1. Supplementary references

**Supplementary methods**

**Animal sampling**

The GJ population is maintained under a predator and rabies control activity of the Israel Nature and Parks Authority (INPA). All specimens were acquired during routine predator control of the INPA and disease surveillance of both INPA and the Kimron Veterinary Institute (KVI) of the Israeli Ministry of Agriculture and Rural Development. GJ sampling was performed as previously described (Lapid et al. 2023) in four different geographical regions in Israel (**Figure S1**): Beit-Shean Valley (1), Ha-Sharon (2), Menashe Heights (3) and the Upper Galilee (4). Beit-Shean Valley and the Upper Galilee are considered “hot spots” for rabies (showing high incidence), while the Menashe Heights (region 3) is considered a moderate hot spot for rabies and Ha-Sharon (region 4) is considered to be free of rabies (Garazi 2018). Sampling and data collection of the specimen occurred immediately after culling of the jackals. The animal’s sex (male/female) and estimated age group (puppy to old) were recorded.

Rectal swabs (ESwab^TM^; Copan Italia S.p.A, Brescia, Italy), and peripheral blood samples (Serum tube- VACUETTE®, Greiner Bio-One, Kremsmünster, Austria; EDTA tube- BD Vacutainer®, BD, Plymouth, UK) were collected from each animal. All samples were stored in a cooler immediately after sampling and transferred within 30-360 minutes to -80^0^C until sample processing. The carcasses were transferred to the KVI for a necropsy and further diagnostic tests.

**Rabies oral vaccination and Toxoplasma tests**

We examined exposure to rabies oral vaccination via tetracycline testing from bone fluorescence (usually mandible); (Who 1989; Yakobson et al. 2008) ; exposure to *Toxoplasma gondii* was assessed using an immunofluorescence antibody test (IFAT) for the detection of specific antibodies (Mazuz et al. 2018) as recently described (Lapid et al. 2023).

**Real-Time quantitative PCR (qPCR)**

DNA was extracted from ESwab fluid using the DNeasy PowerSoil (QIAGEN^®^, Hilden, Germany) kit according to the manufacturer’s instructions. DNA was quantified with the QuBit device (Invitrogen, Waltham, USA). Five singleplex Taqman qPCR assays were used to detect and quantify the following genes; class 1 integron-integrase *Intl1***,** three beta-lactamase genes (*blaCTXM-15, blaSHV, blaTEM1*) and *qnrS* (quinolone resistance). Gene abundance was calculated by normalizing the absolute number of ARG copies to that of 16S *rRNA* gene (Paulus, Hornstra, and Medema 2020). qPCR amplifications were performed in 20ul reactions containing 10ul enzyme mix (2X qPCRBIO probe Mix Hi-ROX, PCR-BIOSYSTEM, London, UK), 600 nmol of each forward and reverse primer and 400 nmol Taqman probe, and 3ul DNA (2ul for 16S *rRNA*). Thermocycling was performed under the following conditions: 95°C for 2 min for initial activation of the DNA polymerase followed by 40 cycles with denaturation at 95°C for 10 sec, annealing and extension at 55°C for 15 and 60°C for 40 sec. The primer and probe sequences are listed in **Table S1**. Assay quality control and quantification the genes *blaCTX-M-15, blaSHV*, and intI1, were achieved using a custom-made sequence cloned into a pUC-GW-Amp plasmid (pMARSALA), while pNORM (Rocha et al. 2020) was used for *blaTEM1*, and *qnrS* genes. In addition, *E.coli* strain ACTT 35218 was used as control for 16S *rRNA* gene.

**Gut microbiome analysis**

Microbial community analysis was performed in R (version 4.3.0) (R. Team 2014) and relied primarily on the tidyverse (Wickham et al. 2019), ggplot2 (Wickham 2009), vegan (Oksanen et al. 2001), and microeco (Liu et al. 2021) packages. We used the *microeco* package in R to normalize samples by total sum scaling. No OTUs were excluded from the dataset to account for background contamination. Relative abundance calculations were performed with the *microeco* package in R. Diversity comparisons were performed by calculating the Shannon Diversity Index (an integrative index of community richness and evenness) (Shannon 1948) and the Bray-Curtis dissimilarity index (a measure of compositional dissimilarity) (Bray and Curtis 1957) using the *vegan* package in R. We determined the significance of differences in community composition using permutational multivariate analysis of variance (PERMANOVA) using the *vegan* package in R. We used a random forest classification model with Mean Decrease in Gini as our index of feature importance to determine the differential abundance of microbial communities and ARGs across samples using the *microeco* package in R.

We used *args_oap* (Yin et al. 2022) to normalize ARGs by the number of 16S *rRNA* reads. We repeated the above microbiome analysis with the output from *args_oap*. We performed a procrustes analysis with the *vegan* package in R to determine the structural correlation between the microbiome at the family level of classification and normalized resistome.

**Bioinformatics analyses**

Taxonomic profiling was performed on the QC reads using kraken2 (v.2.1.2) (Wood, Lu, and Langmead 2019) with the refseq100GB database (Wright, Comeau, and Langille 2023), and the output report for each sample converted to mpa format using taxonkit (v.0.15.0) (Shen and Ren 2021) and collated together with the combine_mpa.py script from KrakenTools (v.1.2) (Lu et al. 2022).

ARGs profiling was performed on the QC reads using ARGs_OAP (v.3.2.3) (Yin et al. 2022). The reads were then assembled using Megahit (https://github.com/voutcn/megahit) (D. Li et al. 2015). The resistome and plasmidome of the assembled MAGs (Metagenome-assembled genomes) were predicted using Abricate (https://github.com/tseemann/abricate), AMRFinderPlus (https://github.com/ncbi/amr) (Feldgarden et al. 2021), the CARD database (Alcock et al. 2023), the MEGARes database (Doster et al. 2019), and the plasmidfinder database (Carattoli et al. 2014).

The MAGs were isolated using SemiBin (https://github.com/BigDataBiology/SemiBin) (Pan et al. 2022) minimap2 (https://github.com/lh3/minimap2) (H. Li 2018), and samtools (https://github.com/samtools/samtools) (Danecek et al. 2021). The taxonomic assignments of the MAGs were determined using GTDBTk (https://github.com/Ecogenomics/GTDBTk) (Chaumeil et al. 2022). The MAGs were quality controlled using CheckM (https://github.com/Ecogenomics/CheckM) (Parks et al. 2015), GUNC (https://github.com/grp-bork/gunc) (Orakov et al. 2021), tRNAscan-SE (https://github.com/UCSC-LoweLab/tRNAscan-SE) (Chan et al. 2021), barRNAp (https://github.com/tseemann/barrnap), and prok-quality (https://github.com/metashot/prok-quality) (Albanese and Donati 2021). The MAGs were dereplicated using dRep (https://github.com/MrOlm/drep) (Olm et al. 2017). The MLST, resistome, and plasmidome of the good quality and dereplicated MAGs were determined using MLST (https://github.com/tseemann/mlst), Abricate (https://github.com/tseemann/abricate), AMRFinderPlus (https://github.com/ncbi/amr), the CARD database (https://github.com/arpcard/amr_curation) , the MEGARes database (https://db.meglab.org/), and the plasmidfinder database (https://cge.cbs.dtu.dk/services/PlasmidFinder/).

**Supplementary Figures**


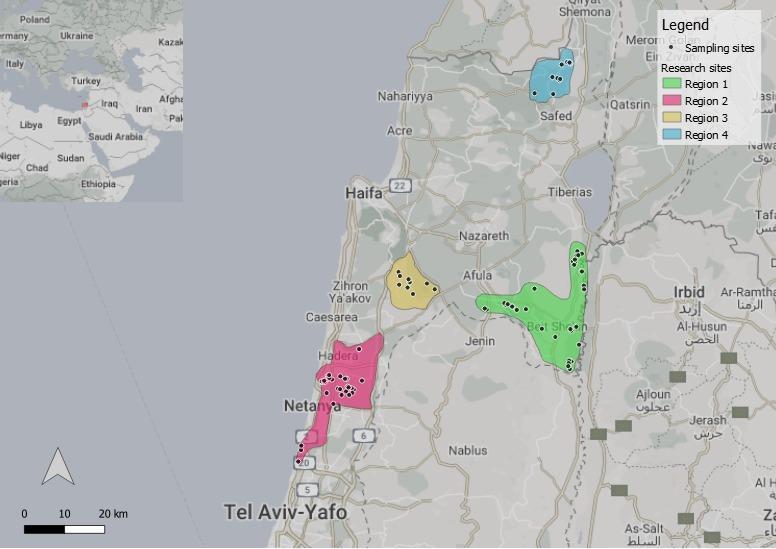


**Figure S1**. Distribution of GJ sampling regions on a map of center-north of Israel: Beit-Shean valley (Region 1; ~347 km^2^), Ha-Sharon (Region 2; ~258 km^2^), Menashe Heights (Region 3; ~115 km^2^) and the Upper Galilee (Region 4; ~100 km^2^)


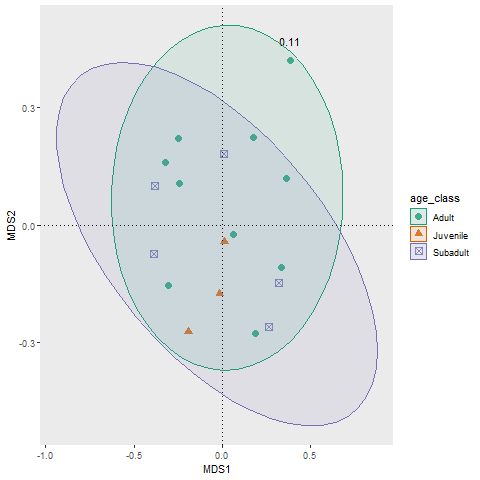
**(A)**


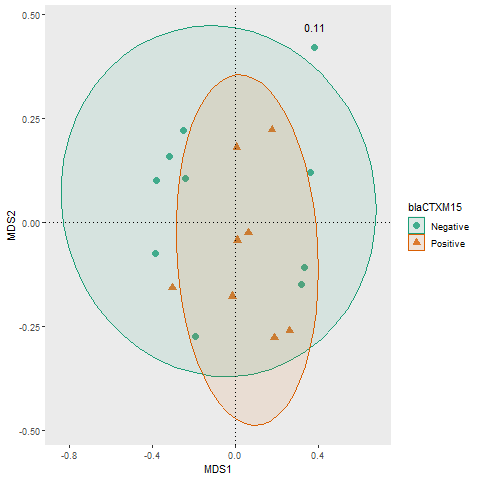
**(B)**

**Figure S2. (A)** NMDS plot demonstrates no discernible difference between *bla*CTXM15 status. **(B)** NMDS plot demonstrates only a discernible difference between juvenile and adult jackal samples.


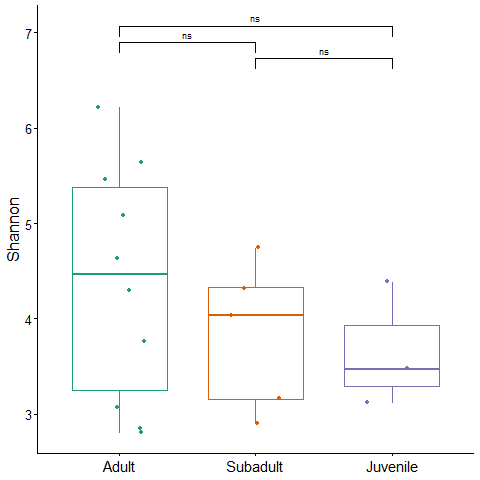


**Figure S3.** Alpha diversity by Shannon index between age classes. Significance was determined with the Wilcoxon Rank Sum test. ns = no significance

**Supplementary tables**

**Table S1:** Primers and probes used in this study

| **Target gene** | **Primer** | **Sequence (5' to 3')** | **Amplicon size** | **Reference** |
| --- | --- | --- | --- | --- |
| ***bla*CTXM-15** | **Cy5** | **Cy5-TCTGGTCACTTACTTCACCCAGCCT- BHQ2** | **103 bp** | **Paulus, 2020** |
|  | ***blaCTXM-15*-F** | **CTATGGCACCACCAACGATA** |  |  |
|  | ***blaCTXM-15*-R** | **ACGGCTTTCTGCCTTAGGTT** |  |  |
| ***bla*SHV** | **FAM** | **FAM-TTGAGCAAATTAAACAAAGCGA- BHQ1** | **768 bp** |  |
|  | ***blaSHV*-F** | **TCGCCTGTGTATTATCTCCC** |  |  |
|  | ***blaSHV*-R** | **TTTAAAGGTGCTCATCATGGGA** |  | **Paulus, 2020 with correction** |
| **Intl1** | **Yakima Yellow** | **YAK-TCGTGATGCCTGCTTGTTCTACGGCA- BHQ1** | **312 bp** | **Paulus, 2020** |
|  | **Intl1-F** | **CGAACGAGTGGCGGAGGGTG** |  |  |
|  | **Intl1-R** | **ACACGCATTCGACCGATC** |  | **Paulus, 2020 with correction** |
| ***qnrS*** | **FAM** | **FAM-TACGACATTCGTCAACTGCAAGT- BHQ1** | **118 bp** | **Paulus, 2020** |
|  | ***qnrS*-F** | **GACGTGCTAACTTGCGTG** |  |  |
|  | ***qnrS*-R** | **TGGCATTGTTGGAAACTT** |  |  |
| ***bla*TEM1** | **Yakima Yellow** | **YAK-TATTCCCTTTTTTGCGGCAT-BHQ1** | **857 bp** |  |
|  | ***blaTEM1*-F** | **GAGTATTCAACATTTTCGT** |  |  |
|  | ***blaTEM1*-R** | **CTCAAGGATCTTACCGCTGTTG** |  | **Paulus, 2020 with correction** |
| **16S *rRNA*** | **Cy5** | **Cy5-CACGAGCTGACGACARCCATGCA- BHQ2** | **193 bp** | **Hoang, 2019** |
|  | **16S rRNA-F** | **TGGAGCATGTGGTTTAATTCGA** |  |  |
|  | **16S rRNA-R** | **TGCGGGACTTAACCCAACA** |  |  |

**Table S2.** General information of the studied GJ cohort

|  | **Region 1**  **(Beit-Shean Valley) (n=40)** | **Region 2**  **(Ha-Sharon)**  **(n=39)** | **Region 3**  **(Menashe Heights)**  **(n=16)** | **Region 4**  **(Upper Galilee)**  **(n=16)** | **Total**  **(n=111)** |
| --- | --- | --- | --- | --- | --- |
| **Female/male ratio** | 21/19 | 20/19 | 9/7 | 7/9 | **57/54** |
| **Female/male ratio (%)** | 52.5/47.5 | 51.28/48.7 | 56.25/43.75 | 43.75/56.25 | **51.35/48.65** |
| **Adult/sub-adult/juvenile ratio** | 21/12/7 | 30/7/2 | 4/11/1 | 8/2/6 | **63/32/16** |
| **Adult/sub-adult/juvenile ratio (%)** | 52.5/30/17.5 | 76.92/17.95/5.13 | 25/68.75/6.25 | 50/12.5/37.5 | **56.76/28.83/14.41** |

**Table S3.** qPCR gene copy number by demographic variables

|  | | ***blaCTX-M-15*** | | | | ***blaSHV*** | | | | ***qnrS*** | | | |
| --- | --- | --- | --- | --- | --- | --- | --- | --- | --- | --- | --- | --- | --- |
|  |  | **mean** | **stdev** | **min** | **max** | **mean** | **stdev** | **min** | **max** | **mean** | **stdev** | **min** | **max** |
| **Region** | 1 (n=40) | 1.52E-12 | 5.19E-12 | 2.27E-23 | 2.26E-11 | 2.97717E-17 | 6.22012E-17 | 3.62E-21 | 1.41E-16 | 2.54E-12 | 1.38E-11 | 1.49E-22 | 7.71E-11 |
|  | 2 (n=39) | 1.73E-15 | 3.21E-15 | 5.81E-23 | 1.12E-14 | 2.94183E-15 | 6.53589E-15 | 4.49E-21 | 1.95E-14 | 1.72E-12 | 6.96E-12 | 7.85E-23 | 3.82E-11 |
|  | 3 (n=16) | 4.11E-15 | 9.95E-15 | 2.78E-24 | 2.86E-14 | 1.30544E-17 | 1.84493E-17 | 8.76E-21 | 2.61E-17 | 4.44E-12 | 1.6E-11 | 4.33E-22 | 5.76E-11 |
|  | 4 (n=16) | 5.74E-15 | 9.89E-15 | 2.95E-25 | 2.47E-14 | #DIV/0! | #DIV/0! | 0 | 0 | 1.15E-12 | 3.59E-12 | 4.23E-19 | 1.25E-11 |
| **Sex** | Male (n=54) | 4.26E-14 | 1.59E-13 | 2.78E-24 | 7.92E-13 | 7.89193E-16 | 2.2025E-15 | 3.62E-21 | 6.24E-15 | 1.45E-12 | 6.31E-12 | 4.33E-22 | 3.82E-11 |
|  | Female (n=57) | 1.18E-12 | 4.67E-12 | 2.95E-25 | 2.26E-11 | 2.54224E-15 | 6.85575E-15 | 4.49E-21 | 1.95E-14 | 3.07E-12 | 1.38E-11 | 7.85E-23 | 7.71E-11 |
| **Age Class** | Adult (n=63) | 1.05E-12 | 4.33E-12 | 2.27E-23 | 2.26E-11 | 2.21849E-15 | 5.72547E-15 | 3.62E-21 | 1.95E-14 | 2.61E-12 | 1.2E-11 | 7.85E-23 | 7.71E-11 |
|  | Sub-Adult (n=32) | 2.38E-15 | 7.89E-15 | 2.78E-24 | 2.86E-14 | 9.54625E-18 | 1.43913E-17 | 8.76E-21 | 2.61E-17 | 2.45E-12 | 1.17E-11 | 4.33E-22 | 5.76E-11 |
|  | Juvenile (n=16) | 1.23E-15 | 3.03E-15 | 2.95E-25 | 8.68E-15 | 9.05E-19 | #DIV/0! | 9.05E-19 | 9.05E-19 | 1.05E-12 | 3.45E-12 | 2.75E-20 | 1.25E-11 |

|  | | ***blaTEM-1*** | | | | ***Intl1*** | | | |
| --- | --- | --- | --- | --- | --- | --- | --- | --- | --- |
|  |  | **mean** | **stdev** | **min** | **max** | **mean** | **stdev** | **min** | **max** |
| **Region** | 1 (n=40) | 1.14E-10 | 6.23E-10 | 1.37E-21 | 3.58E-09 | 1.65E-09 | 9.24E-09 | 9.59E-23 | 5.23E-08 |
|  | 2 (n=39) | 1.12E-11 | 4.93E-11 | 1.87E-32 | 2.85E-10 | 1.1E-11 | 6.1E-11 | 1.25E-22 | 3.45E-10 |
|  | 3 (n=16) | 3.73E-12 | 8.08E-12 | 5.27E-23 | 2.59E-11 | 5.27E-12 | 1.46E-11 | 1.55E-22 | 5.14E-11 |
|  | 4 (n=16) | 8.08E-14 | 1.91E-13 | 2.41E-24 | 5.55E-13 | 5.55E-14 | 1.69E-13 | 6.3E-24 | 6.35E-13 |
| **Sex** | Male (n=54) | 8.9E-11 | 5.4E-10 | 1.87E-32 | 3.58E-09 | 1.26E-09 | 8.07E-09 | 1.55E-22 | 5.23E-08 |
|  | Female (n=57) | 5.84E-12 | 2.22E-11 | 2.41E-24 | 1.49E-10 | 5.89E-12 | 3.3E-11 | 6.3E-24 | 2.31E-10 |
| **Age Class** | Adult (n=63) | 7.7E-11 | 4.88E-10 | 1.87E-32 | 3.58E-09 | 1E-09 | 7.18E-09 | 9.59E-23 | 5.23E-08 |
|  | Sub-Adult (n=32) | 1.61E-12 | 5.42E-12 | 1.52E-22 | 2.59E-11 | 2.08E-12 | 1.03E-11 | 1.55E-22 | 5.14E-11 |
|  | Juvenile (n=16) | 1.15E-12 | 3.99E-12 | 2.41E-24 | 1.6E-11 | 1.88E-12 | 7.09E-12 | 6.3E-24 | 2.75E-11 |

**Table S4. Relative Abundance of Leading Phyla**

| **Phylum** | **Mean (%)** | **Min (%)** | **Max (%)** | **Median (%)** | **IQR (%)** |
| --- | --- | --- | --- | --- | --- |
| Bacteroidota | 46.00 | 14.39 | 75.89 | 47.58 | 34.18 |
| Bacillota | 20.89 | 7.82 | 64.89 | 18.54 | 12.72 |
| Pseudomonadota | 13.34 | 1.43 | 49.08 | 10.08 | 10.75 |
| Campylobacterota | 9.46 | 0.08 | 55.78 | 4.64 | 11.52 |
| Actinomycetota | 4.70 | 1.16 | 18.72 | 2.89 | 2.90 |
| Fusobacteriota | 3.58 | 0.14 | 10.90 | 2.66 | 4.94 |
| Thermodesulfobacteriota | 0.61 | 0.01 | 4.38 | 0.13 | 0.19 |
| Spirochaetota | 0.57 | 0.01 | 7.51 | 0.13 | 0.21 |
| Cyanobacteriota | 0.17 | 0.03 | 0.48 | 0.14 | 0.19 |
| Euryarchaeota | 0.13 | 0.02 | 0.46 | 0.09 | 0.13 |
| **Total** | 99.45 |  |  | 86.88 |  |

**Table S5.** ARGs prevalence in GJ - metagenomics

| **Abundance (%)** | **Gene** | **Antimicrobial class** | **Abundance (%)** | **Gene** | **Antimicrobial class** | **Abundance (%)** | **Gene** | **Antimicrobial class** |
| --- | --- | --- | --- | --- | --- | --- | --- | --- |
| 2.8515 | ugd | **Polymyxin** | 0.0118 | bcr-1 | **Bicyclomycin** | 1.4663 | APH(6)-Id | **Aminoglycoside** |
| 0.6407 | arnA |  | 0.0194 | bleomycin resistance protein | **Bleomycin** | 1.1857 | APH(3'')-Ib |  |
| 0.2203 | pmrF |  | 0.0170 | catI | **Chloramphenicol** | 0.6078 | ANT(6)-Ib |  |
| 0.1825 | rosB |  | 0.0056 | Pseudomonas aeruginos catB7 |  | 0.6064 | aadS |  |
| 0.1590 | eptA |  | 0.0052 | catD |  | 0.1526 | APH(2'')-IIa |  |
| 0.0525 | rosA |  | 0.0041 | catB9 |  | 0.1458 | AAC(6')-Ie-APH(2'')-Ia |  |
| 0.0010 | almG |  | 0.0035 | catA4 |  | 0.0825 | ANT(6)-Ia |  |
| 0.0003 | mcr-3.5 |  | 0.0035 | Vibrio anguillarum chloramphenicol acetyltransferase |  | 0.0644 | AAC(6')-Im |  |
| 0.0002 | mcr-10.1 |  | 0.0027 | catB3 |  | 0.0635 | aad(6) |  |
| 0.0026 | QnrD1 | **Quinolone** | 0.0025 | fexA |  | 0.0627 | APH(3')-IIIa |  |
| 0.0015 | QnrD2 |  | 0.0020 | cmx |  | 0.0603 | APH(2'')-If |  |
| 0.0015 | QnrB5 |  | 0.0011 | Agrobacterium fabrum chloramphenicol acetyltransferase |  | 0.0602 | APH(2'')-IIIa |  |
| 0.0014 | QnrB61 |  | 0.0009 | Pseudomonas aeruginos catB6 |  | 0.0403 | ANT(4')-Ib |  |
| 0.0013 | QnrB54 |  | 0.0009 | Campylobacter coli chloramphenicol acetyltransferase |  | 0.0357 | APH(4)-Ia |  |
| 0.0012 | QnrB30 |  | 0.0005 | catIII |  | 0.0264 | AAC(3)-IV |  |
| 0.0012 | QnrB19 |  | 0.0005 | catB11 |  | 0.0220 | AAC(3)-IId |  |
| 0.0008 | QnrB37 |  | 0.0004 | cmlA5 |  | 0.0211 | aadA |  |
| 0.0008 | QnrB28 |  | 0.0002 | catS |  | 0.0200 | ANT(2'')-Ia |  |
| 0.0007 | QnrB70 |  | 0.1572 | Clostridium perfringens mprF | **Defensin** | 0.0119 | aadA5 |  |
| 0.0007 | QnrS8 |  | 0.0452 | floR | **Florfenicol** | 0.0111 | APH(3')-Ia |  |
| 0.0005 | QnrB21 |  | 0.0029 | pp-flo |  | 0.0099 | aadA3 |  |
| 0.0004 | QnrS7 |  | 0.0026 | FosA | **Fosfomycin** | 0.0095 | aadA8 |  |
| 0.0003 | QnrB25 |  | 0.0007 | FosA6 |  | 0.0078 | AAC(3)-IIe |  |
| 0.0003 | QnrB27 |  | 0.0005 | FosA7 |  | 0.0050 | aadA12 |  |
| 0.0003 | QnrB38 |  | 0.0003 | FosA5 |  | 0.0043 | APH(3')-IIb |  |
| 0.0003 | QnrS5 |  | 0.0002 | FosA3 |  | 0.0040 | aadA22 |  |
| 0.0002 | QnrB33 |  | 11.6842 | mef(En2) | **Macrolide-lincosamide-streptogramin** | 0.0030 | aadA2 |  |
| 0.0002 | QnrS4 |  | 7.7337 | mel |  | 0.0024 | aadA8b |  |
| 0.0002 | norA |  | 1.4279 | lnu(C) |  | 0.0022 | aadA23 |  |
| 0.0002 | norB |  | 1.0911 | erm(F) |  | 0.0015 | aadA14 |  |
| 0.0002 | QnrB23 |  | 1.0264 | optrA |  | 0.0013 | ANT(9)-Ia |  |
| 0.0002 | QnrB10 |  | 0.8560 | lsa(B) |  | 0.0011 | aadA25 |  |
| 0.0002 | QnrB50 |  | 0.3564 | macB |  | 0.0009 | aadK |  |
| 0.0002 | QnrB66 |  | 0.1547 | mph(B) |  | 0.0009 | aadA24 |  |
| 0.0002 | QnrB72 |  | 0.1175 | erm(Q) |  | 0.0009 | aadA13 |  |
| 0.0001 | QnrB71 |  | 0.1026 | macA |  | 0.0005 | AAC(6')-Ib7 |  |
| 0.0001 | QnrB8 |  | 0.0657 | LlmA 23S ribosomal RNA methyltransferase |  | 0.0005 | aadA21 |  |
| 0.0001 | QnrB12 |  | 0.0561 | erm(B) |  | 0.0005 | Other streptomycin resistance protein |  |
| 0.0001 | QnrB57 |  | 0.0522 | vat(B) |  | 0.0004 | aadA17 |  |
| 0.0001 | QnrB60 |  | 0.0229 | poxtA |  | 0.0004 | aadA27 |  |
| 0.0001 | QnrB68 |  | 0.0157 | erm(G) |  | 0.0002 | AAC(6')-Iaj |  |
| 0.0001 | QnrB73 |  | 0.0132 | clcD |  | 0.0002 | AAC(2')-Ia |  |
| 0.0038 | rphB | **Rifamycin** | 0.0117 | mph(A) |  | 0.0001 | amrB |  |
| 0.0011 | rphA |  | 0.0116 | cfr(B) |  | 0.0036 | antibacterial_fatty_acid__farA | **Antibacterial_fatty_acid** |
| 0.0009 | arr-3 |  | 0.0062 | cipA |  | 0.0006 | antibacterial_fatty_acid__farB |  |
| 0.0007 | arr-2 |  | 0.0049 | lmrD |  | 1.4100 | bacA | **Bacitracin** |
| 0.0004 | arr-5 |  | 0.0039 | lnu(P) |  | 0.0286 | bcrA |  |
| 0.0001 | RbpA |  | 0.0034 | cfr(E) |  | 1.5602 | CfxA6 | **Beta_lactam** |
| 0.1009 | SAT-4 | **Streptothricin** | 0.0029 | lsa(A) |  | 0.6987 | CfxA2 |  |
| 0.0124 | SatA |  | 0.0027 | cfr(C) |  | 0.2902 | OXA-347 |  |
| 0.0009 | SAT-2 |  | 0.0022 | cfr(A) |  | 0.2286 | CfxA5 |  |
| 1.4768 | sul2 | **Sulfonamide** | 0.0021 | lsa(E) |  | 0.1886 | Escherichia coli ampC |  |
| 0.1323 | sul1 |  | 0.0018 | erm(X) |  | 0.1826 | CfxA3 |  |
| 0.0007 | sul3 |  | 0.0015 | mef(B) |  | 0.1222 | Klebsiella pneumoniae OmpK37 |  |
| 24.7918 | tet(Q) | **Tetracycline** | 0.0012 | vat(E) |  | 0.1150 | OXA-85 |  |
| 7.4075 | tet(O) |  | 0.0010 | lnu(E) |  | 0.0726 | OXA-209 |  |
| 5.3159 | tet(W) |  | 0.0007 | lnu(B) |  | 0.0687 | CfxA4 |  |
| 4.4383 | tet(32) |  | 0.0006 | lnu(D) |  | 0.0470 | CblA-1 |  |
| 3.3420 | tet(40) |  | 0.0006 | ere(D) |  | 0.0464 | CfxA |  |
| 1.1264 | tet(M) |  | 0.0006 | erm(47) |  | 0.0460 | TEM-117 |  |
| 0.9893 | tetA(P) |  | 0.0005 | lnu(G) |  | 0.0296 | TEM-1 |  |
| 0.8851 | tet(A) |  | 0.0004 | vat(A) |  | 0.0277 | cepA |  |
| 0.7693 | tet(G) |  | 0.0003 | vga(C) |  | 0.0148 | ACI-1 |  |
| 0.7207 | tetX2 |  | 0.0001 | VatI |  | 0.0127 | OXA-460 |  |
| 0.6243 | tetB(P) |  | 0.0001 | erm(39) |  | 0.0121 | PDC-10 |  |
| 0.3515 | tet(O/W) |  | 0.0001 | mef(E) |  | 0.0117 | CcrA beta-lactamase |  |
| 0.3512 | tet(W/N/W) |  | 0.6082 | RanA | **Efflux** | 0.0085 | TEM-193 |  |
| 0.3400 | tet(X3) |  | 0.4184 | msbA |  | 0.0065 | TEM-192 |  |
| 0.2670 | tet(44) |  | 0.2972 | mdtM |  | 0.0060 | Other class C beta-lactamase |  |
| 0.2527 | tet(X) |  | 0.2887 | mdtK |  | 0.0043 | TEM-7 |  |
| 0.1099 | tet(X5) |  | 0.2672 | emrD |  | 0.0040 | OXA-486 |  |
| 0.0867 | tet(L) |  | 0.2438 | Escherichia coli mdfA |  | 0.0040 | OXA-50 |  |
| 0.0810 | tetX6 |  | 0.2368 | RanB |  | 0.0028 | OXA-576 |  |
| 0.0783 | tet(X4) |  | 0.2356 | mdtL |  | 0.0027 | OXA-192 |  |
| 0.0652 | tet(37) |  | 0.2031 | mdtF |  | 0.0027 | PDC-88 |  |
| 0.0431 | tet(35) |  | 0.2017 | tolC |  | 0.0026 | OXA-63 |  |
| 0.0424 | tet(H) |  | 0.1848 | acrF |  | 0.0024 | TEM-118 |  |
| 0.0344 | tet(Y) |  | 0.1711 | mdtE |  | 0.0022 | TEM-87 |  |
| 0.0164 | tet(B) |  | 0.1546 | MexB |  | 0.0022 | TEM-63 |  |
| 0.0157 | tet(61) |  | 0.1525 | mdtH |  | 0.0020 | VIM-1 |  |
| 0.0081 | tet(34) |  | 0.1387 | mdtP |  | 0.0019 | PDC-5 |  |
| 0.0075 | tet(J) |  | 0.1262 | acrE |  | 0.0017 | TEM-178 |  |
| 0.0070 | tet(58) |  | 0.1175 | emrB |  | 0.0013 | OXA-450 |  |
| 0.0024 | tet(43) |  | 0.1124 | sdeY |  | 0.0013 | PDC-1 |  |
| 0.0021 | tet(D) |  | 0.1121 | emrA |  | 0.0012 | PDC-3 |  |
| 0.0014 | tet(T) |  | 0.1119 | mdtO |  | 0.0011 | TEM-136 |  |
| 0.0011 | tet(C) |  | 0.1074 | emrK |  | 0.0010 | TEM-184 |  |
| 0.0011 | tet(Z) |  | 0.0935 | efrB |  | 0.0009 | ACT-30 |  |
| 0.0005 | tet(K) |  | 0.0925 | Escherichia coli acrA |  | 0.0009 | LAP-2 |  |
| 0.0005 | tetA(60) |  | 0.0924 | emrY |  | 0.0008 | OXA-449 |  |
| 0.0004 | tet(E) |  | 0.0767 | smeE |  | 0.0008 | TEM-91 |  |
| 0.0003 | tet(S) |  | 0.0654 | mdtN |  | 0.0007 | MOX-7 |  |
| 0.0002 | tetB(46) |  | 0.0613 | MuxB |  | 0.0007 | ACT-17 |  |
| 0.0387 | dfrF | **Trimethoprim** | 0.0393 | fexB |  | 0.0007 | OXA-451 |  |
| 0.0061 | dfrA17 |  | 0.0367 | MuxC |  | 0.0007 | PDC-81 |  |
| 0.0043 | dfrA12 |  | 0.0354 | Escherichia coli emrE |  | 0.0006 | CMH-1 |  |
| 0.0032 | dfrA14 |  | 0.0185 | cmeC |  | 0.0006 | ACT-20 |  |
| 0.0016 | dfrA1 |  | 0.0168 | mexW |  | 0.0006 | CTX-M-123 |  |
| 0.0009 | dfrA5 |  | 0.0149 | MexD |  | 0.0006 | OXA-61 |  |
| 0.0008 | dfrG |  | 0.0125 | Other_major_facilitator_superfamily_transporter |  | 0.0005 | CMY-82 |  |
| 0.0004 | dfrA3 |  | 0.0115 | qacEdelta1 |  | 0.0005 | OXA-448 |  |
| 0.0094 | vanG | **Vancomycin** | 0.0111 | mexK |  | 0.0005 | TEM-75 |  |
| 0.0073 | vanYG1 |  | 0.0099 | PmpM |  | 0.0004 | CMY-2 |  |
| 0.0008 | vanF |  | 0.0097 | mexQ |  | 0.0004 | CMY-45 |  |
| 0.0008 | vanXD |  | 0.0095 | ParS |  | 0.0004 | CMY-73 |  |
| 0.0006 | vanTN |  | 0.0091 | MexE |  | 0.0004 | CMY-83 |  |
| 0.0006 | vanYA |  | 0.0090 | MexF |  | 0.0004 | CMY-99 |  |
| 0.0004 | vanB |  | 0.0072 | oqxB |  | 0.0004 | DHA-1 |  |
| 0.0004 | vanHB |  | 0.0071 | mexN |  | 0.0004 | DHA-19 |  |
| 0.0004 | vanTG |  | 0.0067 | efrA |  | 0.0004 | TEM-148 |  |
| 0.0003 | vanA |  | 0.0066 | mexH |  | 0.0004 | TEM-33 |  |
| 0.0003 | vanD |  | 0.0064 | OprM |  | 0.0004 | Yrc-1 |  |
| 0.0002 | vanM |  | 0.0056 | OpmH |  | 0.0004 | ACT-15 |  |
| 0.0002 | vanO |  | 0.0054 | mexG |  | 0.0004 | ACT-16 |  |
| 0.0002 | vanXF |  | 0.0050 | opmD |  | 0.0004 | PDC-7 |  |
| 0.0002 | vanZF |  | 0.0045 | OprJ |  | 0.0004 | PDC-83 |  |
| 0.0001 | vanXO |  | 0.0040 | MexA |  | 0.0004 | PDC-92 |  |
|  |  |  | 0.0037 | Pseudomonas aeruginosa emrE |  | 0.0003 | Bla1 |  |
|  |  |  | 0.0037 | qacE |  | 0.0003 | DHA-5 |  |
|  |  |  | 0.0032 | opmE |  | 0.0003 | DHA-9 |  |
|  |  |  | 0.0031 | MexC |  | 0.0003 | OXA-1 |  |
|  |  |  | 0.0030 | mexV |  | 0.0003 | TEM-105 |  |
|  |  |  | 0.0028 | mexJ |  | 0.0003 | TEM-108 |  |
|  |  |  | 0.0026 | OpmB |  | 0.0003 | TEM-12 |  |
|  |  |  | 0.0025 | OprN |  | 0.0003 | TEM-123 |  |
|  |  |  | 0.0025 | efpA |  | 0.0003 | TEM-131 |  |
|  |  |  | 0.0024 | MuxA |  | 0.0003 | TEM-143 |  |
|  |  |  | 0.0021 | mexM |  | 0.0003 | TEM-195 |  |
|  |  |  | 0.0020 | mexP |  | 0.0003 | TEM-6 |  |
|  |  |  | 0.0020 | smeD |  | 0.0003 | TEM-78 |  |
|  |  |  | 0.0020 | oqxA |  | 0.0003 | SHV-22 |  |
|  |  |  | 0.0019 | hmrM |  | 0.0002 | SHV-167 |  |
|  |  |  | 0.0013 | patB |  | 0.0002 | ACT-7 |  |
|  |  |  | 0.0012 | cdeA |  | 0.0002 | CTX-M-19 |  |
|  |  |  | 0.0012 | bmr |  | 0.0002 | CTX-M-27 |  |
|  |  |  | 0.0012 | mtrD |  | 0.0002 | OXA-461 |  |
|  |  |  | 0.0011 | efmA |  | 0.0002 | OXA-472 |  |
|  |  |  | 0.0009 | smeF |  | 0.0002 | OXA-485 |  |
|  |  |  | 0.0008 | emeA |  | 0.0002 | OXA-488 |  |
|  |  |  | 0.0008 | hp1181 |  | 0.0002 | PDC-2 |  |
|  |  |  | 0.0008 | Klebsiella pneumoniae acrA |  | 0.0002 | PDC-77 |  |
|  |  |  | 0.0008 | Enterobacter cloacae acrA |  | 0.0002 | PDC-79 |  |
|  |  |  | 0.0005 | qacH |  | 0.0002 | PDC-82 |  |
|  |  |  | 0.0003 | patA |  | 0.0002 | PDC-93 |  |
|  |  |  | 0.0002 | mdsB |  | 0.0002 | TEM-121 |  |
|  |  |  | 0.0002 | mtrE |  | 0.0002 | TEM-128 |  |
|  |  |  | 0.0002 | adeF |  | 0.0002 | BPU-1 |  |
|  |  |  | 0.2468 | mupA | **Mupirocin** | 0.0002 | TEM-59 |  |
|  |  |  | 0.1098 | mupB |  | 0.0002 | TEM-70 |  |
|  |  |  | 0.0982 | Bifidobacteria intrinsic ileS conferring resistance to mupirocin |  | 0.0001 | ACT-28 |  |
|  |  |  | 0.0032 | novA | **Novobiocin** | 0.0001 | MIR-14 |  |
|  |  |  | 0.2442 | microcin efflux pumu gene yojI | **Other_peptide_antibiotics** | 0.0001 | CMY-101 |  |
|  |  |  | 0.0510 | ArnT |  | 0.0001 | OXY-2-1 |  |
|  |  |  | 0.1468 | TaeA | **Pleuromutilin_tiamulin** | 0.0001 | OXY-2-4 |  |
|  |  |  | 0.0231 | tva(A) |  | 0.0001 | VHH-1 |  |
|  |  |  |  |  |  | 0.0001 | TEM-153 |  |
|  |  |  |  |  |  | 0.0001 | TEM-182 |  |
|  |  |  |  |  |  | 0.0001 | TEM-88 |  |
|  |  |  |  |  |  | 0.0001 | SHV-105 |  |

**Table S6**. Concordance between detection of ARGs with qPCR and metagenomic methods (Yellow- not detected, Blue- detected)

| **qPCR** | **MGx** | **qPCR** | **MGx** | **qPCR** | **MGx** | **qPCR** | **MGx** |  |
| --- | --- | --- | --- | --- | --- | --- | --- | --- |
| **CTX-M** | **CTX-M** | **TEM** | **TEM** | **SHv** | **SHv** | **qnrS** | **qnrS** | **Jackal ID** |
|  |  |  |  |  |  |  |  | **R109** |
|  |  |  |  |  |  |  |  | **R11** |
|  |  |  |  |  |  |  |  | **R110** |
|  |  |  |  |  |  |  |  | **R111** |
|  |  |  |  |  |  |  |  | **R112** |
|  |  |  |  |  |  |  |  | **R13** |
|  |  |  |  |  |  |  |  | **R19** |
|  |  |  |  |  |  |  |  | **R20** |
|  |  |  |  |  |  |  |  | **R22** |
|  |  |  |  |  |  |  |  | **R23** |
|  |  |  |  |  |  |  |  | **R32** |
|  |  |  |  |  |  |  |  | **R37** |
|  |  |  |  |  |  |  |  | **R42** |
|  |  |  |  |  |  |  |  | **R67** |
|  |  |  |  |  |  |  |  | **R73** |
|  |  |  |  |  |  |  |  | **R76** |
|  |  |  |  |  |  |  |  | **R83** |

**Table S7.** Taxa recovered by MLST

| **Taxon** | **uniqueID** | **GJ** | **MLST-scheme** | **ST number** | **Loci1** | **Loci2** | **Loci3** | **Loci4** | **Loci5** | **Loci6** | **Loci7** | **Loci8** |
| --- | --- | --- | --- | --- | --- | --- | --- | --- | --- | --- | --- | --- |
| Bacteroides fragilis | R22_env.dog_gut_bin.66 | R22 | bfragilis | - | dnaJ(4) | fusA(-) | groL(5) | prfA(47) | recA(3) | rpoB(1) | rprX(-) |  |
| Brachyspira | R13_env.dog_gut_bin.150 | R13 | brachyspira | - | Bra_adh(~16) | Bra_alp(-) | Bra_est(~13) | Bra_gdh(20) | Bra_glp(~21) | Bra_pgm(~15) | Bra_thi(29) |  |
| Campylobacter_D coli | R83_env.dog_gut_bin.59 | R83 | campylobacter | 1770 | aspA(33) | glnA(39) | gltA(30) | glyA(82) | pgm(104) | tkt(206) | uncA(17) |  |
| Campylobacter_D upsaliensis | R20_env.dog_gut_bin.90 | R20 | campylobacter_nonjejuni_4 | - | Cup_adk(-) | Cup_aspA(12) | Cup_atpA(1) | Cup_glnA(10) | Cup_glyA(36?) | Cup_pgi(1) | Cup_tkt(9) |  |
| Clostridium_P perfringens | R110_env.dog_gut_bin.2 | R110 | cperfringens | - | colA(~44) | groEL(~17) | sodA(136?) | plc(17) | gyrB(12) | sigK(13) | pgk(~7) | nadA(~15) |
| Escherichia coli | R19_env.dog_gut_bin.64 | R19 | ecoli_achtman_4 | - | adk(6) | fumC(6) | gyrB(15) | icd(~56) | mdh(11) | purA(26) | recA(6) |  |
| Escherichia coli | R37_env.dog_gut_bin.1 | R37 | ecoli_achtman_4 | - | adk(6) | fumC(29) | gyrB(33) | icd(16) | mdh(11) | purA(8) | recA(2) |  |
| Escherichia coli | R76_env.dog_gut_bin.13 | R76 | ecoli_achtman_4 | - | adk(10) | fumC(185) | gyrB(~488) | icd(8) | mdh(~642) | purA(18) | recA(2) |  |

**Supplementary references**

1. Albanese, Davide, and Claudio Donati. 2021. “Large-Scale Quality Assessment of Prokaryotic Genomes with Metashot/Prok-Quality.” *F1000Research* 10 (August): 822.
2. Alcock, Brian P., William Huynh, Romeo Chalil, Keaton W. Smith, Amogelang R. Raphenya, Mateusz A. Wlodarski, Arman Edalatmand, et al. 2023. “CARD 2023: Expanded Curation, Support for Machine Learning, and Resistome Prediction at the Comprehensive Antibiotic Resistance Database.” *Nucleic Acids Research* 51 (D1): D690–99.
3. Bray, J. Roger, and J. T. Curtis. 1957. “An Ordination of the Upland Forest Communities of Southern Wisconsin.” *Ecological Monographs* 27 (4): 325–49.
4. Carattoli, Alessandra, Ea Zankari, Aurora García-Fernández, Mette Voldby Larsen, Ole Lund, Laura Villa, Frank Møller Aarestrup, and Henrik Hasman. 2014. “In Silico Detection and Typing of Plasmids Using PlasmidFinder and Plasmid Multilocus Sequence Typing.” *Antimicrobial Agents and Chemotherapy* 58 (7): 3895–3903.
5. Chan, Patricia P., Brian Y. Lin, Allysia J. Mak, and Todd M. Lowe. 2021. “TRNAscan-SE 2.0: Improved Detection and Functional Classification of Transfer RNA Genes.” *Nucleic Acids Research* 49 (16): 9077–96.
6. Chaumeil, Pierre-Alain, Aaron J. Mussig, Philip Hugenholtz, and Donovan H. Parks. 2022. “GTDB-Tk v2: Memory Friendly Classification with the Genome Taxonomy Database.” *Bioinformatics*  38 (23): 5315–16.
7. Danecek, Petr, James K. Bonfield, Jennifer Liddle, John Marshall, Valeriu Ohan, Martin O. Pollard, Andrew Whitwham, et al. 2021. “Twelve Years of SAMtools and BCFtools.” *GigaScience* 10 (2). https://doi.org/10.1093/gigascience/giab008.
8. Doster, Enrique, Steven M. Lakin, Christopher J. Dean, Cory Wolfe, Jared G. Young, Christina Boucher, Keith E. Belk, Noelle R. Noyes, and Paul S. Morley. 2019. “MEGARes 2.0: A Database for Classification of Antimicrobial Drug, Biocide and Metal Resistance Determinants in Metagenomic Sequence Data.” *Nucleic Acids Research* 48 (D1): D561–69.
9. Feldgarden, Michael, Vyacheslav Brover, Narjol Gonzalez-Escalona, Jonathan G. Frye, Julie Haendiges, Daniel H. Haft, Maria Hoffmann, et al. 2021. “AMRFinderPlus and the Reference Gene Catalog Facilitate Examination of the Genomic Links among Antimicrobial Resistance, Stress Response, and Virulence.” *Scientific Reports* 11 (1): 12728.
10. Garazi, S. 2018. “Rabies, Follow-up Report No. 1 (Final Report).” https://www.oie.int/wahis_2/public/wahid.php/Reviewreport/Review?page_refer=MapFullEventReport&reportid=26204.
11. Lapid, Roi, Yair Motro, Hillary Craddock, Boris Khalfin, Roni King, Gila Kahila Bar-Gal, and Jacob Moran-Gilad. 2023. “Fecal Microbiota of the Synanthropic Golden Jackal (Canis Aureus).” *Animal Microbiome* 5 (1): 37.
12. Li, Dinghua, Chi-Man Liu, Ruibang Luo, Kunihiko Sadakane, and Tak-Wah Lam. 2015. “MEGAHIT: An Ultra-Fast Single-Node Solution for Large and Complex Metagenomics Assembly via Succinct de Bruijn Graph.” *Bioinformatics*  31 (10): 1674–76.
13. Li, Heng. 2018. “Minimap2: Pairwise Alignment for Nucleotide Sequences.” *Bioinformatics*  34 (18): 3094–3100.
14. Liu, Chi, Yaoming Cui, Xiangzhen Li, and Minjie Yao. 2021. “Microeco: An R Package for Data Mining in Microbial Community Ecology.” *FEMS Microbiology Ecology* 97 (2). https://doi.org/10.1093/femsec/fiaa255.
15. Lu, Jennifer, Natalia Rincon, Derrick E. Wood, Florian P. Breitwieser, Christopher Pockrandt, Ben Langmead, Steven L. Salzberg, and Martin Steinegger. 2022. “Metagenome Analysis Using the Kraken Software Suite.” *Nature Protocols* 17 (12): 2815–39.
16. Mazuz, Monica L., Gema Alvarez-García, Roni King, Igor Savisky, Varda Shkap, Luis M. Ortega-Mora, and Daniel Gutiérrez-Expósito. 2018. “Exposure to Neospora Spp. and Besnoitia Spp. in Wildlife from Israel.” *International Journal for Parasitology. Parasites and Wildlife* 7 (3): 317–21.
17. Oksanen, Jari, Gavin L. Simpson, F. Guillaume Blanchet, Roeland Kindt, Pierre Legendre, Peter R. Minchin, R. B. O’Hara, et al. 2001. “Vegan: Community Ecology Package.” *CRAN: Contributed Packages*. The R Foundation. https://doi.org/10.32614/cran.package.vegan.
18. Olm, Matthew R., Christopher T. Brown, Brandon Brooks, and Jillian F. Banfield. 2017. “DRep: A Tool for Fast and Accurate Genomic Comparisons That Enables Improved Genome Recovery from Metagenomes through de-Replication.” *The ISME Journal* 11 (12): 2864–68.
19. Orakov, Askarbek, Anthony Fullam, Luis Pedro Coelho, Supriya Khedkar, Damian Szklarczyk, Daniel R. Mende, Thomas S. B. Schmidt, and Peer Bork. 2021. “GUNC: Detection of Chimerism and Contamination in Prokaryotic Genomes.” *Genome Biology* 22 (1): 178.
20. Pan, Shaojun, Chengkai Zhu, Xing-Ming Zhao, and Luis Pedro Coelho. 2022. “A Deep Siamese Neural Network Improves Metagenome-Assembled Genomes in Microbiome Datasets across Different Environments.” *Nature Communications* 13 (1): 2326.
21. Parks, Donovan H., Michael Imelfort, Connor T. Skennerton, Philip Hugenholtz, and Gene W. Tyson. 2015. “CheckM: Assessing the Quality of Microbial Genomes Recovered from Isolates, Single Cells, and Metagenomes.” *Genome Research* 25 (7): 1043–55.
22. Paulus, Gabriela K., Luc M. Hornstra, and Gertjan Medema. 2020. “International Tempo-Spatial Study of Antibiotic Resistance Genes across the Rhine River Using Newly Developed Multiplex QPCR Assays.” *The Science of the Total Environment* 706: 135733.
23. R. Team. 2014. “R: A Language and Environment for Statistical Computing.” *MSOR Connections* 1. https://apps.dtic.mil/sti/citations/AD1039033.
24. Rocha, Jaqueline, Damiano Cacace, Ioannis Kampouris, Hélène Guilloteau, Thomas Jäger, Roberto B. M. Marano, Popi Karaolia, et al. 2020. “Inter-Laboratory Calibration of Quantitative Analyses of Antibiotic Resistance Genes.” *Journal of Environmental Chemical Engineering* 8 (1): 102214.
25. Shannon, C. E. 1948. “A Mathematical Theory of Communication.” *The Bell System Technical Journal* 27 (3): 379–423.
26. Shen, Wei, and Hong Ren. 2021. “TaxonKit: A Practical and Efficient NCBI Taxonomy Toolkit.” *Journal of Genetics and Genomics = Yi Chuan Xue Bao* 48 (9): 844–50.
27. Who. 1989. “Requirements and Criteria for Field Trials on Oral Rabies Vaccination of Dogs and Wild Carnivores.” https://apps.who.int/iris/bitstream/handle/10665/58394/WHO_Rab.Res_89.32.pdf?sequence=1&isAllowed=y.
28. Wickham, Hadley. 2009. *Ggplot2: Elegant Graphics for Data Analysis*. Springer Science & Business Media.
29. Wickham, Hadley, Mara Averick, Jennifer Bryan, Winston Chang, Lucy McGowan, Romain François, Garrett Grolemund, et al. 2019. “Welcome to the Tidyverse.” *Journal of Open Source Software* 4 (43): 1686.
30. Wood, Derrick E., Jennifer Lu, and Ben Langmead. 2019. “Improved Metagenomic Analysis with Kraken 2.” *Genome Biology* 20 (1): 257.
31. Wright, Robyn J., Andrè M. Comeau, and Morgan G. I. Langille. 2023. “From Defaults to Databases: Parameter and Database Choice Dramatically Impact the Performance of Metagenomic Taxonomic Classification Tools.” *Microbial Genomics* 9 (3). https://doi.org/10.1099/mgen.0.000949.
32. Yakobson, B. A., R. King, N. Sheichat, B. Eventov, and D. David. 2008. “Assessment of the Efficacy of Oral Vaccination of Livestock Guardian Dogs in the Framework of Oral Rabies Vaccination of Wild Canids in Israel.” *Developments in Biologicals* 131: 151–56.
33. Yin, Xiaole, Xiawan Zheng, Liguan Li, An-Ni Zhang, Xiao-Tao Jiang, and Tong Zhang. 2022. “ARGs-OAP v3.0: Antibiotic-Resistance Gene Database Curation and Analysis Pipeline Optimization.” *Proceedings of the Estonian Academy of Sciences: Engineering*, December. https://doi.org/10.1016/j.eng.2022.10.011.
